# Expression of CPPED1 in human trophoblasts is associated with timing of term birth

**DOI:** 10.1111/jcmm.13402

**Published:** 2017-11-29

**Authors:** Antti M. Haapalainen, Minna K. Karjalainen, Ravindra Daddali, Steffen Ohlmeier, Julia Anttonen, Tomi A. Määttä, Annamari Salminen, Mari Mahlman, Ulrich Bergmann, Kaarin Mäkikallio, Marja Ojaniemi, Mikko Hallman, Mika Rämet

**Affiliations:** ^1^ PEDEGO Research Unit and Medical Research Center Oulu University of Oulu Oulu Finland; ^2^ Department of Children and Adolescents Oulu University Hospital Oulu Finland; ^3^ Proteomics Core Facility Biocenter Oulu Faculty of Biochemistry and Molecular Medicine University of Oulu Oulu Finland; ^4^ Department of Obstetrics and Gynecology Oulu University Hospital Oulu Finland; ^5^ Department of Obstetrics and Gynecology Turku University Hospital and University of Turku Turku Finland; ^6^ BioMediTech Institute and Faculty of Medical and Life Sciences University of Tampere Tampere Finland

**Keywords:** CPPED1, genetics, placenta, proteomics, trophoblast

## Abstract

Understanding of timing of human parturition is incomplete. Therefore, we carried out proteomic analyses of full‐term placentas from uncomplicated pregnancies to identify protein signatures associated with the onset of spontaneous delivery. We found quantitative associations of 10 proteins with spontaneous term birth, evident either in the basal or in the chorionic plates or in both. Additional 18 proteins were associated according to the location within placenta indicating local variations in protein amounts. Calcineurin‐like phosphoesterase domain‐containing 1 (CPPED1), a phosphatase previously suggested dephosphorylating AKT1/PKB, was one of the identified proteins. qRT‐PCR revealed the mRNA level of *CPPED1* was higher in elective caesarean deliveries than in spontaneous births, while immunohistochemistry showed CPPED1 in cytotrophoblasts, syncytiotrophoblasts and extravillous trophoblasts. Noteworthy, phosphorylation status of AKT1 did not differ between placentas from elective caesarean and spontaneous deliveries. Additionally, analyses of samples from infants indicated that single‐nucleotide polymorphisms rs11643593 and rs8048866 of *CPPED1* were associated with duration of term pregnancy. Finally, post‐transcriptional silencing of *CPPED1* in cultured HTR8/SVneo cells by siRNAs affected gene expression in pathways associated with inflammation and blood vessel development. We postulate that functions regulated by CPPED1 in trophoblasts at choriodecidual interphase have a role in the induction of term labour, but it may be independent of AKT1.

## Introduction

Despite extensive research, our understanding of the timing of human parturition at the molecular level remains incomplete. Multiple factors may initiate labour 1, and the signal can originate from the mother, foetus, uteroplacental unit or a combination of these. Some hormones are known to influence the maintenance of pregnancy. These include steroid hormones such as progesterone, oestrogens, and androgens, as well as the peptide hormones chorionic gonadotropin, chorionic somatomammotropin, relaxin and oxytocin, which further interact with the immune system to promote pregnancy 2, 3. Progesterone is essential for uterine quiescence; oestrogen, on the other hand, increases myometrial sensitivity to oxytocin, thereby inducing contractions 2. Strong evidence from animal studies also suggests that the foetal hypothalamic–pituitary–adrenal (HPA) axis controls secretion of corticotrophin‐releasing hormone (CRH), leading to increased induction of foetal maturity by corticosteroids and increased secretion of labour‐inducing hormones 4. In the placenta, CRH expression is negatively regulated by progesterone and oestrogen and positively regulated by various neuropeptides 1, 5, 6, 7. Other known factors, such as uterine stretch, inflammatory cytokines, prostaglandins and nitric oxide, have known or proposed roles in the initiation of labour.

CRH and foetal fibronectin (FFN) are additional key proteins that have been used to monitor the timing of parturition in humans 1. In maternal plasma, CRH levels increase exponentially during pregnancy 1. Because there are large individual variations, the rate of increase rather than a single CRH measurement should be determined 1, 8. By contrast, FFN is present mainly in amniotic fluid and foetal membranes and in placental tissue. FFN may be released into cervicovaginal secretions through mechanical and infection‐mediated damage to the foetal membranes or placenta prior to birth 9. Elevated FFN levels in cervical fluids indicate an increased likelihood of delivery; thus, they are a predictor of term and preterm birth 9, 10. Unlike in experimental animals such as sheep and small rodents, in human pregnancy blood levels of progesterone increase rather than decrease during labour. It has been proposed that functional withdrawal of progesterone upon labour in humans may occur, as expression of a low‐affinity variant of progesterone receptor increases towards term 11. In human placenta, trophoblasts produce and secrete progesterone 12.

Some studies have reported crosstalk between progesterone and oestrogen receptors and RAC‐alpha serine/threonine‐protein kinase (AKT1) in various diseases 13. AKT1 is part of the phosphatidylinositol‐3‐kinase (PI3K) signalling pathway 14, 15, and in some cancers, the PI3K/AKT pathway is hyperactivated 13. Phosphorylation of Ser473 is required for full activation of AKT1; this is accomplished by 3‐phosphoinositide‐dependent protein kinase 2 (PDK2) 16, 17, 18. Calcineurin‐like phosphoesterase domain‐containing 1 (CPPED1) is a newly characterized phosphatase. It inactivates AKT1 by removing the critical phosphate from Ser473 of AKT1 19.

Recent epigenetic, transcriptomic, proteomic and exosome analyses of human placenta and the placental microbiome have revealed new data, not only about placental development but also about labour. Placenta‐derived exosomes, released to the maternal circulation, appear to have roles in regulating diverse functions, including placental development and immunotolerance 20. Placental transcriptomic differences have been identified in the decidua, amnion and chorion, suggesting various functionally distinct subregions 21, 22. Moreover, epigenetic variation in the placenta may be associated with pregnancy outcome 23.

Several proteomic studies have reported biomarkers of term and preterm births 10, 24, 25. Over a hundred biomarkers of spontaneous preterm birth have been identified for various types of biochemical pathways 26. More than half of these biomarkers have been classified as being involved in immune function and inflammation. Proteomic analyses of placental blood plasma 27 and cervicovaginal fluids 28 from normal term deliveries have identified proteins linked to immune and defence responses and inflammation and oxidative stress, respectively. Proteomic analyses of placenta have also focused on foetal growth restriction 29, early pregnancy loss 30, 31, 32, pre‐eclampsia 32, 33, down syndrome 34, differences between first‐ and third‐trimester human placentas 35 and low molecular weight components in the chorionic and basal plates of placenta 36.

The majority of placental studies have focused on different pathological conditions, while only few proteomic studies have investigated changes associated with normal term birth. The aim of this study was to identify placental proteins associated with gestational age and labour in uncomplicated term pregnancies. To do so, we analysed the proteome of the chorionic and basal plates of human placenta to identify proteins involved in the normal labour process. We found that the extraembryonic protein CPPED1 is not only a promising marker for spontaneous term birth but that a polymorphism of the *CPPED1* gene is associated with the length of normal uncomplicated pregnancy. We also provide evidence that in human term placenta, CPPED1 levels are not associated with the phosphorylation status of AKT1.

## Materials and methods

### Placenta samples

The study was approved by the ethics committee of Oulu University Hospital, and all mothers provided written informed consent. Placenta tissues were collected at Oulu University Hospital in 2012–2014. Samples were harvested as described previously 37, including tissue biopsies from both basal and chorionic plates. All placental specimens were from uncomplicated term pregnancies. Of the cases (*n *=* *31), 18 were spontaneous vaginal deliveries and 13 infants were delivered electively by Caesarean section without signs or symptoms of labour. Of the spontaneous term deliveries, the gestational age (GA) varied from 39 weeks + 6 days to 41 weeks + 3 days (average 40 week + 1 day GA). In the elective caesarean term birth group, the placentas were from pregnancies with GAs of 38 weeks + 3 days to 42 weeks + 0 day (average 39 weeks + 2 days GA). The length of pregnancy was based on foetal ultrasound examination before 16 weeks of pregnancy.

### Two‐dimensional minimal difference gel electrophoresis and mass spectrometry

For the proteomic study, we included placenta samples collected after either elective caesarean or spontaneous term birth from the chorionic and basal plates (*n *=* *6 per group). For protein spots to be considered significant, we used a ± 1.5‐fold cut‐off ratio. A *P* value of ≤0.05 according to Student's *t*‐test was considered statistically significant. Theoretical spot positions in the 2D gel according to known protein sequences were calculated with the Compute pI/Mw tool (http://ca.expasy.org/tools/pi_tool.html). Associations between proteomic changes and GO biological processes and molecular functions were identified with the Database for Annotation, Visualization and Integrated Discovery (DAVID), v 6.7 38, 39. In mass spectrometry, to identify proteins, we ran additional 2D gels with larger amounts of unlabelled protein (400–800 μg) combined with Cyanine dye (Cy) 2‐labelled internal standard. Further details regarding 2D‐gel and MS analyses are presented in the Appendix S1.

### Study population for the genetic study of gestational age

The study population was collected prospectively at Oulu University Hospital in 2004–2007 and 2014. Altogether, we included 342 infants born spontaneously at term (GA from 38 weeks + 0 days to 41 weeks + 6 days; GA mean ± standard deviation [S.D.] 40.1 ± 0.9 weeks). All study subjects were of Finnish origin. Umbilical cord blood or umbilical cord tissue was collected for DNA extraction. Specific inclusion criteria are presented in the Appendix S1.

### Genetic study and genotyping

Genes encoding proteins that we identified as up‐ or down‐regulated in the placental proteome from spontaneous term deliveries were analysed for their association with GA. Altogether, we investigated 77 SNPs: *ACTB*, three SNPs; *A2M*, nine SNPs; *B2M*, one SNP; *CPPED1*, 16 SNPs; *CYB5A*, 11 SNPs; *HBG2*, five SNPs; *KRT8*, 12 SNPs; *KRT19*, five SNPs; *PRDX2*, three SNPs; and *SERPINB2*, 12 SNPs (Table S4). Associations between these SNPs and GA were assessed by the Wald test with PLINK, v. 1.07 40. Under strict Bonferroni correction, the significance level was *P *<* *6.5 × 10^−4^.

We focused in depth on genotypes of the two GA‐associated *CPPED1* SNPs rs11643593 and rs8048866 in the investigated infants. Further details are presented in the Appendix S1.

### Western blotting

To quantitate the amount of AKT1 phosphorylated at Ser473 compared to the total amount of AKT1, we used rabbit anti‐human AKT1 pS473 antibody (2967S, 1:1000 dilution; Cell Signaling Technology, Leiden, The Netherlands) and mouse anti‐human AKT1 antibody (9018S, 1:1000 dilution; Cell Signaling Technology), respectively. Secondary antibodies were goat anti‐rabbit IgG Dylight 680 conjugate (611‐144‐002‐0.5, 1:10000 dilution; Rockland, Limerick, PA, USA) and goat antimouse IgG Dylight 800 conjugate (610‐145‐002‐0.5, 1:10000 dilution; Rockland). Detection was done with the Odyssey Infrared Imaging System (LI‐COR Biosciences, Lincoln, NE, USA). We used 87 ng of human recombinant AKT1 (009‐001‐P21; Rockland) as a positive control.

To quantitate the amount of FOXO1 phosphorylated at Ser256 compared to the total amount of FOXO1, we used rabbit anti‐human FoxO1 pSer256 antibody (NB100‐81927, 1:1000 dilution; Novus Biologicals, Abingdon, Oxon, UK) and mouse anti‐human FOXO1 antibody (F6928, 1:1000 dilution; Sigma‐Aldrich, St. Louis, MO, USA), respectively. The secondary antibody and detection method were the same as for AKT1. Further details are presented in the Appendix S1.

### Immunohistochemistry

For CPPED1 detection, samples were incubated in rabbit anti‐human CPPED1 antibody (HPA040938, 1:250 dilution; Sigma‐Aldrich) for 1 hr, followed by detection of the bound antibodies with the two‐step Envision kit (K5007; DAKO). For FOXO1 and FOXO3 detection, samples were incubated in rabbit anti‐human phospho‐FOXO1 pSer256 antibody (SAB4300094, 1:4000 dilution; Sigma‐Aldrich) and rabbit anti‐human phospho‐FOXO3 pSer253 antibody (PA5‐37578, 1:10000 dilution; Thermo Fisher Scientific, Waltham, MA, USA), respectively. For negative controls, non‐immune rabbit IgG was used to confirm the specificity of the primary antibody. Further details are presented in the Appendix S1.

### Quantitative PCR

We included samples from the basal and chorionic plates of placentas from 13 elective caesarean and 18 spontaneous term births for qRT‐PCR analysis. RNA isolation, cDNA synthesis and quantitative PCR analyses were done as described earlier 37. The High Pure RNA Tissue Kit (Roche, Branchburg, NJ, USA) and RNeasy Micro Kit (Qiagen, Hilden, Germany) were used for RNA isolation. Relative quantifications of *CPPED1* mRNA levels from chorionic and basal plates were done with the LightCycler^®^96 instrument (Roche). The experiments were designed as intron spanning assays with *cytochrome c1 (CYC1)* mRNA as a reference. Further details regarding quantitative PCR are presented in the Appendix S1.

### Transfection with small interfering RNAs

HTR8/SVneo cells (ATCC, CRL‐3271) were initially transfected with either 30 nM of negative control siRNA or 100 nM of pooled *CPPED1* siRNAs (siRNA pairs 1–3) in suspensions and then added into 12‐well plate. After 24 hrs of incubation, adherent cells were re‐transfected with siRNA pairs 1–3 at the same concentration as transfection performed in suspension and cells harvested after 48 hrs of second transfection. Further details regarding transfection are presented in the Appendix S1.

### Transcriptomes of siRNA samples

Prior to transcriptomic analysis of siRNA samples, the RNA quality was determined by Agilent 2100 Bioanalyzer system at Biocenter Oulu Sequencing Center, Finland. The transcriptomes of the *CPPED1* silenced cells and negative control cells were determined using Illumina HiSeq high‐throughput sequencing system at the Finnish Functional Genomics Centre, Finland. The sequencing data were analysed by the Bioinformatics Unit Core Service at the Turku Centre for Biotechnology, Finland.

## Results

### Protein signatures associated with spontaneous term deliveries

To explore the molecular mechanisms associated with spontaneous term delivery, we compared protein quantities in human placentas from uncomplicated pregnancies with spontaneous and elective caesarean term deliveries. We likewise investigated differences within the placenta proteome by studying tissue samples collected from basal and chorionic plates. Gel‐based proteomics with difference gel electrophoresis (DIGE) approach was used to study not only protein levels but also modification and processing of intact proteins. In the first step, sample preparation, protein labelling and separation were optimized for placenta tissue, which resulted in the reproducible detection of about 2,400 spots (Fig. S1). The applied software for two‐dimensional (2D) gel analysis allows 100% spot matching; therefore, all spots were compared among the four study groups, which comprised spontaneous or elective caesarean birth with samples collected from the basal and chorionic plates of the same placenta (*n *=* *6 per group). Comparisons of both delivery types as well as placenta locations revealed 49 significantly changed spots (cut‐off ratio: ±1.5‐fold, *P *≤* *0.05; Fig. S1), which were identified by mass spectrometry (MS) analyses to correspond with 28 proteins (Tables 1 and S1).

**Table 1:** Birth phenotype‐ and location‐dependent changes in the human placenta proteome

Spot	Protein	UniProt‐KB	Description	Theoretical pI/MW (kD)	Detected pI/MW (kD)	Ratio			
						E *versus* S		Ba *versus* Ch	
						Ba	Ch	E	S
Elective (E) *versus* spontaneous (S) birth									
46*	ACTB	P60709	Actin, cytoplasmic 1 (fragment)	5.29/41.7 (5.29/41.6)	4.93 18	1,57	(1,13)	2,17	(1,54)
1	A2M	P01023	α‐2‐macroglobulin	6.03/163.3	5.51 142	−1,57	(−1,45)	(1,12)	(1,22)
2				(5.98/160.8)	5.54 142	−1,62	−1,59	(1,09)	(1,13)
3					5.57 142	−1,55	−1,67	(−1,04)	(−1,11)
49	B2M	P61769	β‐2‐microglobulin	6.06/13.7 (6.07/11.7)	6.07 14	(1,12)	1,59	(−1,45)	(−1,04)
39	CPPED1	Q9BRF8	Serine/threonine‐protein phosphatase (isoform 1)	5.79/35.5	5.75 31	−1,71	−1,79	(−1,14)	(−1,19)
44	CYB5A	P00167	Cytochrome b5 (isoform 1 or 2)	4.86/15.3^1^	4.72 18	−1,74	−1,59	(1,07)	(1,17)
47	HBG2	P69892	Haemoglobin subunit γ‐2	6.64/16.1	6.71 16	−2,57	−2,26	(1,03)	(1,18)
48				(6.71/15.9)	6.54 16	−2,33	−2,03	(1,13)	(1,29)
13	KRT8	P05787	Keratin, type II cytoskeletal 8 (isoform 1 or 2)	5.52/53.7^1^	5.24 49	(1,16)	1,71	(−1,14)	(1,30)
14			isoform 1 or 2	5.52/53.7^1^	5.24 47	(1,08)	1,73	(−1,29)	(1,24)
15			isoform 1 or 2	5.52/53.7^1^	5.33 50	(1,55)	2,09	(1,09)	(1,46)
30*			fragment of isoform 1 or 2	5.52/53.7	4.79 37	(1,81)	2,39	(1,59)	(2,09)
31*			fragment of isoform 1 or 2	5.52/53.7	4.85 38	(1,88)	2,77	(1,07)	(1,56)
21	KRT19	P08727	Keratin, type I cytoskeletal 19	5.05/44.2	4.85 41	(−1,09)	1,59	(−1,15)	(1,48)
29*			C‐terminal KRT19 fragment	5.05/44.2	4.74 37	(1,19)	1,62	(1,12)	(1,54)
42	PRDX2	P32119	Peroxiredoxin‐2 (isoform 1)	5.66/21.9 (5.67/21.8)	5.52 22	−1,65	(−1,49)	(1,10)	(1,21)
22	SERPINB2	P05120	Plasminogen activator inhibitor 2	5.46/46.6	5.51 42	(−1,14)	−1,92	(−1,18)	(−1,99)
Basal plate (Ba) *versus* chorionic plate (Ch)									
46*	ACTB	P60709	Actin, cytoplasmic 1 (fragment)	5.29/41.7 (5.29/41.6)	4.93 18	1,57	(1,13)	2,17	(1,54)
38	ANXA3	P12429	Annexin A3	5.62/36.4 (5.63/36.2)	5.58 31	(−1,35)	(1,39)	(1,52)	2,83
45*	ANXA5	P08758	Annexin A5 (fragment)	4.93/35.9 (4.93/35.8)	4,83 18	(1,39)	(−1,04)	1,55	(1,08)
41	APCS	P02743	Serum amyloid P‐component	6.10/25.4 (6.12/25.3)	5.52 25	(1,13)	(1,67)	(1,89)	2,75
32	CLU	P10909	Clusterin (isoform 3 or fragments of isoforms 1,2,4, 5)	5.88/52.5^1^	4.76 36	(1,13)	(1,65)	(1,98)	2,87
33			isoform 3 or fragments of isoforms 1,2,4, 5	(5.89/50.1^1^)	4.82 35	(1,13)	(1,57)	2,16	2,98
34			isoform 3 or fragments of isoforms 1,2,4, 5		4.88 34	(−1,11)	(1,38)	2,43	3,69
5	EEF2	P13639	Elongation factor 2	6.41/95.3 (6.42/95.2)	6.42 95	(−1,02)	(−1,77)	(−1,34)	−2,32
23*	FGB	P02675	Fibrinogen β (C‐terminal fragment in D dimer)	8.54/55.9	5.51 38	(1,43)	(1,57)	2,25	2,45
24*			C‐terminal fragment in D dimer	(7.95/50.8)	5.60 39	(1,28)	(1,63)	2,49	3,17
25*			C‐terminal fragment in D dimer		5.71 39	(1,16)	(1,56)	2,54	3,42
26*			C‐terminal fragment in D dimer		5.85 38	(1,56)	(1,61)	1,73	(1,81)
6	FGG	P02679	Fibrinogen γ (isoform gamma‐A or –B)	5.37/51.5^A^	5.46 94	(2,55)	(1,68)	2,07	(1,36)
7			isoform γ‐A or –B	(5.24/48.5^A^)	5.54 94	(2,18)	(1,56)	2,15	(1,53)
16			isoform γ‐A or –B		5.54 50	(−1,13)	(1,16)	1,58	2,05
43	FTL	P02792	Ferritin light chain	5.50/20.0 (5.50/19.9)	5.50 20	(−1,03)	(−1,47)	(−1,97)	−2,75
4	GSN	P06396	Gelsolin (isoform 1)	5.90/85.7 (5.72/82.9)	5.82 95	(−1,58)	(1,37)	(1,19)	2,59
40	HIBADH	P31937	3‐hydroxyisobutyrate dehydrogenase	8.38/35.3 (5.54/31.5)	5.62 29	(−1,01)	(−1,42)	(−1,15)	−1,65
11	HNRNPK	P61978	Heterogeneous nuclear ribonucleoprotein K (isoform 1, 2 or 3)	5.39/50.9^1^	5.39 56	(1,27)	(−1,74)	(−1,10)	−2,45
27	HSD17B1	P14061	Estradiol 17‐β‐dehydrogenase 1	5.46/34.9	5.46 35	(1,11)	(−1,40)	(−1,25)	−1,96
28				(5.47/34.8)	5.59 35	(1,09)	(−1,43)	(−1,32)	−2,05
10	LMNB1	P20700	Lamin‐B1	5.11/66.4 (5.11/65.9)	5.11 66	(−1,01)	(−1,36)	(−1,13)	−1,53
17	LUM	P51884	Lumican	6.16/38.4	4.45 53	(−1,21)	(1,48)	2,81	5,02
18				(6.17/36.7)	4.51 53	(−1,24)	(1,52)	(2,38)	4,52
19					4.61 53	(−1,29)	(2,22)	(2,65)	7,59
20					4.71 52	(−1,01)	(2,34)	(2,25)	5,23
36	TPM1	P09493	Tropomyosin α‐1 chain (isoform 8)	4.71/32.8	4.71 33	(−1,06)	(1,36)	2,10	3,03
37			isoform 1 or 9	4.69/32.7^1^	4.69 33	(−1,09)	(1,32)	(2,01)	2,90
35	TPM2	P07951	Tropomyosin β chain (isoform 2)	4.63/32.9	4.62 35	(−1,04)	(1,54)	2,45	3,91
8	VPS35	Q96QK1	Vacuolar protein sorting‐associated protein 35	5.32/91.7	5.36 82	(−1,02)	(−1,91)	(−1,60)	−2,99
9					5.41 82	(1,02)	(−1,54)	(−1,38)	−2,17
12	WARS	P23381	Tryptophan‐tRNA ligase (isoform 1 or 2)	5.83/53.2^1^ (5.83/53.0^1^)	5.83 55	(1,09)	(−1,45)	(−1,42)	−2,29

Spot numbers are according to Fig. S1. Descriptions and UniProt accession numbers for the identified spots are shown. Spots belonging to the same protein are clustered. Protein fragments are indicated with asterisks. Specific protein isoforms were validated according to spot positions in the gel and the covered protein sequence as well as isoform‐specific peptides obtained by mass spectrometry. Theoretical spot identifications were calculated according to full or mature (in parentheses) protein sequences, whereas detected spot positions in the gel were determined according to selected marker spots. If the identified spot allowed for the presence of several isoforms, theoretical values were indicated for the most common isoform 1 or A. Ratio shows the change in mean normalized spot volumes between elective (E) and spontaneous (S) term birth or between basal (Ba) and chorionic (Ch) plates of the placenta. Changes with a too‐low ratio (<1.5‐fold) or no significance (*P *>* *0.05) are denoted by parentheses. Only the amount of one protein (ACTB) changed significantly in both comparisons (E *versus* S and Ba *versus* Ch). For more details about protein levels, statistical significance and protein identification, see Tables S1 and S2.

**Figure 1:**
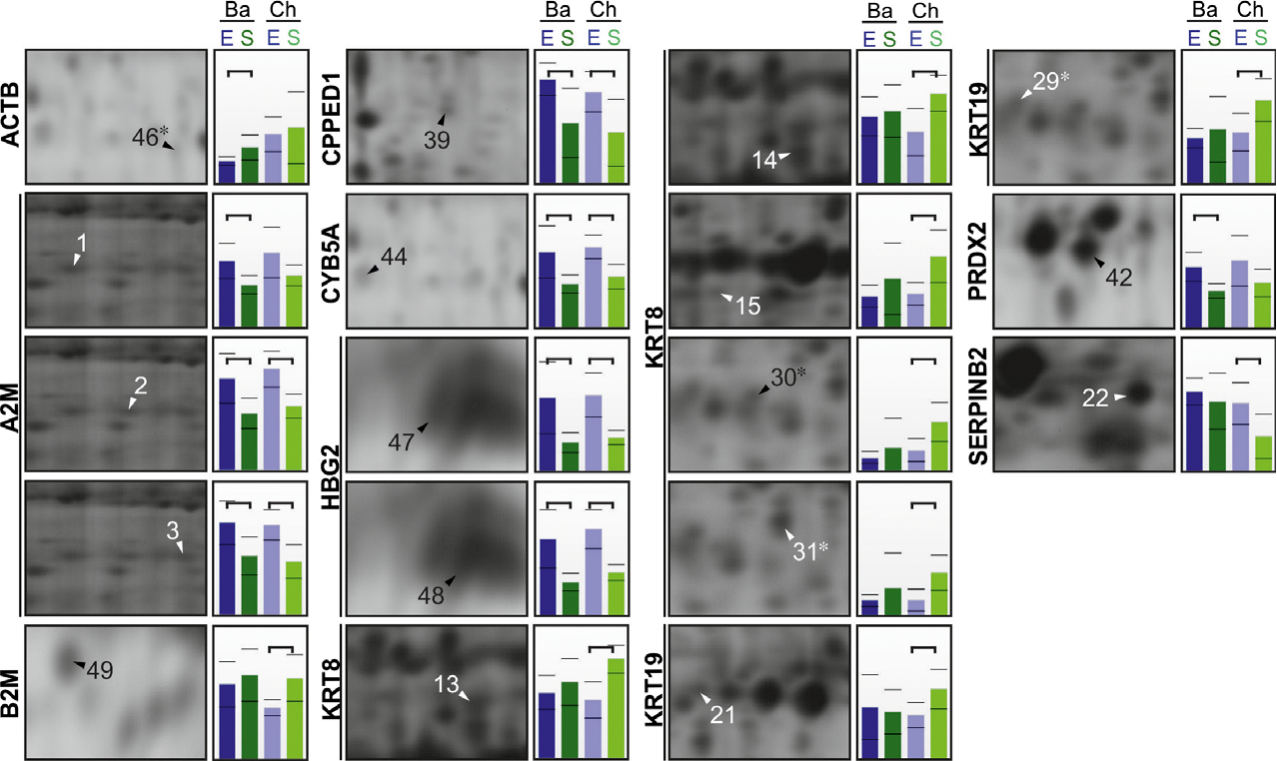
Proteomic changes between elective (E) and spontaneous (S) term birth in basal (Ba) and chorionic (Ch) plates of the placenta. Sections of the gel represent the exact spot positions and expression profiles of the protein level according to the mean normalized spot volumes for the spots shown in Fig. S1. Spots corresponding to the same protein are clustered with a vertical line. Protein fragments are indicated with asterisks. Statistically significant changes (*P *<* *0.05) in expression profiles are shown by horizontal brackets. In the comparisons, six placenta samples were included per group.

Ten proteins changed based on the mode of delivery (Fig 1; Table 1). Spontaneous delivery compared to elective caesarean birth was associated with up‐regulation of four proteins: ACTB, β‐2‐microglobulin (B2M), keratin type II cytoskeletal 8 (KRT8) and 19 (KRT19). Six proteins were down‐regulated in spontaneous delivery compared to elective caesarean birth: α‐2‐macroglobulin (A2M), CPPED1, cytochrome b5 (CYB5A), haemoglobin subunit γ‐2 (HBG2), peroxiredoxin‐2 (PRDX2) and plasminogen activator inhibitor 2 (SERPINB2). We detected statistically significant differences for A2M, CPPED1, CYB5A and HGB2 in both the chorionic and the basal plates. By contrast, ACTB and PRDX2 were only significantly changed in the basal plate and B2M, KRT8, KRT19 and SERPINB2 were only significantly changed in the chorionic plate (Fig. 1).

### Different proteomic profiles in placental chorionic and basal plates

**Figure 2:**
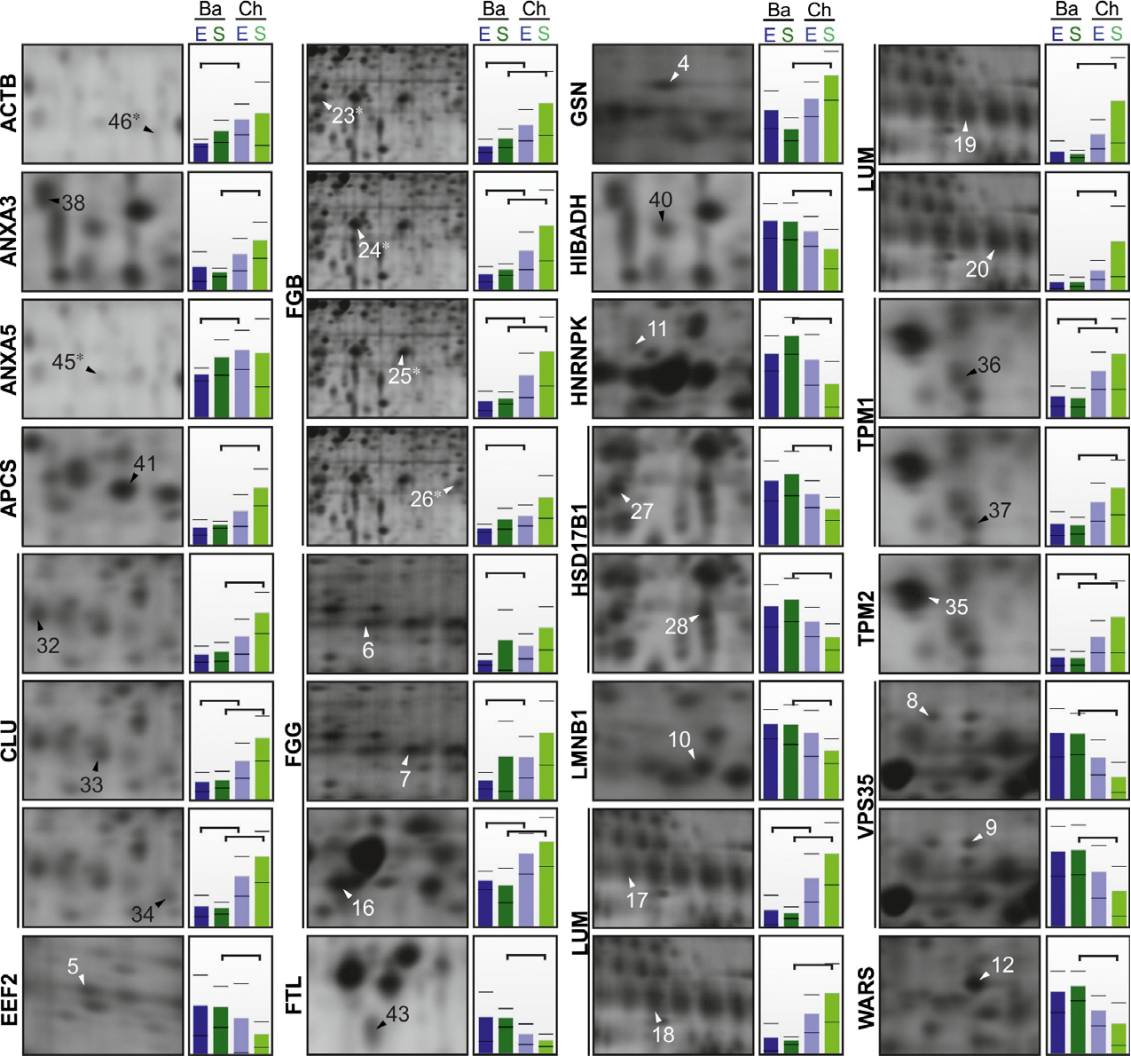
Proteomic changes between basal (Ba) and chorionic (Ch) plates of the placenta. Samples were collected after elective (E) or spontaneous (S) term birth. Gel parts represent the exact spot positions and expression profiles of the protein level according to the mean normalized spot volumes for the spots shown in Fig. S1. For spots 17–20, the exact positions are shown in gel parts representative of the chorionic plate. Spots corresponding to the same protein are clustered with a vertical line. Protein fragments are indicated with asterisks. Statistically significant changes (*P *<* *0.05) in expression profiles are shown by horizontal brackets. In the comparisons, six placenta samples were included per group.

We took samples of placenta from two opposite locations within the same tissue (chorionic and basal plates) to additionally determine whether there were local variations in protein levels independent of birth phenotype. Totally, 19 proteins were altered based on location in the chorionic and basal plates of the same placenta after spontaneous and elective caesarean term delivery (*n *=* *6 per group; Fig. 2; Table 1).

We found higher protein levels in the chorionic plate compared to the basal plate for 11 proteins: ACTB, annexin A3 (ANXA3), annexin A5 (ANXA5), serum amyloid P‐component (APCS), clusterin (CLU), fibrinogen β (FGB), fibrinogen γ (FGG), gelsolin (GSN), lumican (LUM), tropomyosin α‐1 chain (TPM1) and tropomyosin β chain (TPM2). The change was statistically significant after spontaneous and elective caesarean delivery for CLU, FGB, FGG, LUM and TPM1 and 2. ACTB and ANXA5 were significantly changed after elective caesarean delivery, and ANXA3, APCS and GSN were significantly changed after spontaneous delivery.

Eight proteins were present at lower levels in the chorionic plate compared to the basal plate: elongation factor 2 (EEF2), ferritin light chain (FTL), 3‐hydroxyisobutyrate dehydrogenase (HIBADH), heterogeneous nuclear ribonucleoprotein K (HNRNPK), estradiol 17‐β‐dehydrogenase 1 (HSD17B1), lamin‐B1 (LMNB1), vacuolar protein sorting‐associated protein 35 (VPS35) and tryptophan‐tRNA ligase (WARS). These changes were only significant after spontaneous delivery (Fig. 2; Table 1). Our findings indicate that there are functionally different subregions within the placenta, highlighting the importance of carefully collecting samples from the same location.

### Delivery type and tissue location alter specific protein variants

We detected several proteins in multiple spots and/or at unexpected positions in the 2D gel. This suggests that additional regulatory mechanisms such as alternative splicing, protein processing, fragmentation and other modifications are taking place. Therefore, the theoretical spot positions calculated with Compute pI/Mw and the detected spot positions in the gel (Fig. S1; Table 1) were compared. In addition, sequence coverage and spot‐specific peptides obtained by MS (Table S2) were also examined. We found specific isoforms associated with the delivery type (*e.g*. CPPED1 isoform 1 and PRDX2 isoform 1) and placenta location (*e.g*. GSN isoform 1, TPM1 isoform 8 and TPM2 isoform 2). Likewise, numerous protein fragments were associated with delivery type (KRT8 and KRT19), placenta location (ANXA5 and FGB) or both (ACTB). However, all multiple spots of A2M, HBG2, KRT8, KRT19, as well as CLU, FGB, FGG, HSD17B1, LUM, TPM1 and VPS35 showed changes similar to those of the corresponding protein. Thus, the detected protein signatures are likely a result of altered gene expression rather than specific processing.

### Protein changes during spontaneous term delivery mostly associated with immune system and structural/cytoskeletal organization

We further clustered proteins associated with the delivery type (Table 1) according to their gene ontology (GO) biological processes and molecular functions (Table S3). The majority of the GO terms were linked to either the immune system or to structural/cytoskeletal organization. Biological processes comprising at least three changed proteins included responses to inorganic substance (ACTB, A2M and PRDX2), organic substance (A2M, B2M, KRT19 and PRDX2) and wounding (A2M, PRDX2 and SERPINB2). Structural molecule activity (ACTB, KRT8 and KRT19) represented the major molecular function. In addition, A2M and SERPINB2 were in the same blood coagulation pathway. CPPED1 was not associated with any of the enriched GO terms. As a phosphatase, CPPED1 is thought to be involved in blocking cell cycle progression, promoting apoptosis and affecting glucose metabolism 19, 41.

### *CPPED1* polymorphism associated with gestational age

Changes in protein levels are expected during labour. To identify proteins that are important in initiating labour rather than affected by it, we investigated whether single‐nucleotide polymorphisms (SNPs) within 20 kb of the genes encoding the aforementioned proteins (*ACTB*,* A2M*,* B2M*,* CPPED1*,* CYB5A*,* HBG2*,* KRT8*,* KRT19*,* PRDX2* and *SERPINB2*) were associated with gestational age (GA) in infants born at term. We analysed 77 SNPs from 342 samples from infants born between 38 and 42 weeks of gestation. Of all the analysed SNPs, only two intronic SNPs within *CPPED1* were associated with GA at the Bonferroni‐corrected significance level (*P *<* *6.5 × 10^−4^): rs11643593 (*P *=* *2.7 × 10^−4^) and rs8048866 (*P *=* *3.3 × 10^−4^; Table S4). The minor allele of rs11643593 (A) and major allele of rs8048866 (A) were associated with higher GA (Table S5). This suggests that CPPED1 may be involved in the initiation of term delivery and prompted us to study its expression in the placenta during labour in more detail.

### CPPED1 expressed in trophoblasts of full‐term placentas

**Figure 3:**
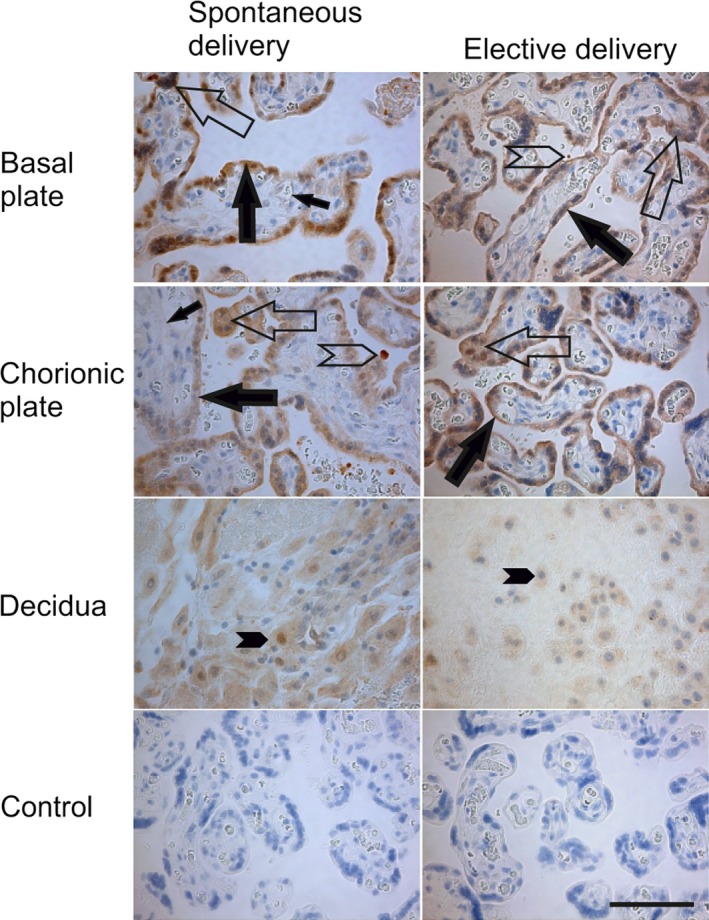
Placental localization of CPPED1 in spontaneous and elective births. Immunostaining of CPPED1 in full‐term placentas from spontaneous (*n *=* *6) or elective births (*n *=* *6). Representative placentas immunostained with anti‐human CPPED1 antibodies. Immunostaining is indicated by filled large arrows in cytotrophoblasts, unfilled large arrows in syncytiotrophoblasts, filled small arrows in endothelium, unfilled arrowheads in leucocytes and filled arrowheads in decidual trophoblasts. Original magnification is 20× in all figures. Control represents isotype controls for immunostaining. Scale bar, 100 μm.

To determine which cell types express CPPED1 in the placenta, we immunostained placentas from spontaneous and elective caesarean term deliveries with anti‐human CPPED1 antibody. As shown in Figure 3, a CPPED1 signal was obtained from samples of both basal and chorionic plates of spontaneous and elective term placentas. CPPED1 was detected in the cytoplasm and cell membranes of cytotrophoblasts and syncytiotrophoblasts (Fig. 3). CPPED1 staining was also positive in the epithelium of the amniotic membranes. In the decidua, extravillous trophoblasts were positive for CPPED1. The capillary endothelia of the villi showed only faint and patchy staining, whereas intracapillary and intervillous leucocytes were highly positive (Fig. 3).

### Lower levels of *CPPED1* mRNA in spontaneous term deliveries

**Figure 4:**
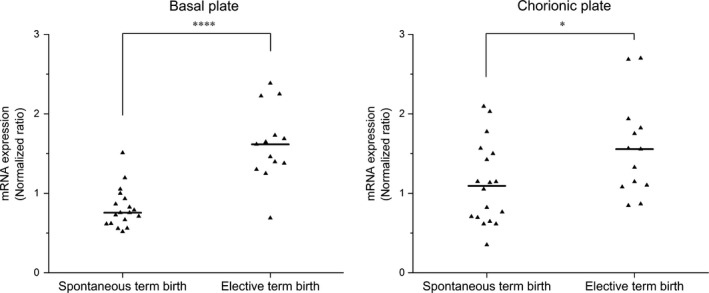
The mRNA levels of *CPPED1* in spontaneous and elective term placentas. Relative mRNA levels from the basal or chorionic plates were normalized to mRNA levels of the housekeeping gene *CYC1*. Differences were analysed with non‐parametric Mann–Whitney *U*‐test. Statistically significant changes according to the non‐parametric Mann–Whitney *U*‐test are indicated by one (*P *<* *0.05) and four (*P *<* *0.0001) asterisks. Solid lines denote median values.

To investigate whether the decreased levels of CPPED1 protein that we observed in spontaneous delivery were the result of altered transcription, we performed quantitative PCR (qPCR) analysis. Figure 4 shows a comparison of *CPPED1* mRNA levels after spontaneous (*n *=* *18) and elective caesarean (*n *=* *13) deliveries in the basal and chorionic plates of the placenta. *CPPED1* mRNA levels were significantly lower in spontaneous term deliveries compared to elective caesarean term births in the basal (2.3‐fold, *P *=* *0.000062) and chorionic (1.5‐fold, *P *=* *0.041) plates. The genotypes of the two *CPPED1* SNPs (rs11643593 and rs8048866) associated with GA in term infants were not significantly associated with mRNA levels. However, the change in *CPPED1* mRNA levels was in line with what we observed for CPPED1 protein levels.

### CPPED1 does not affect phosphorylation of AKT1 and FOXO1

**Figure 5:**
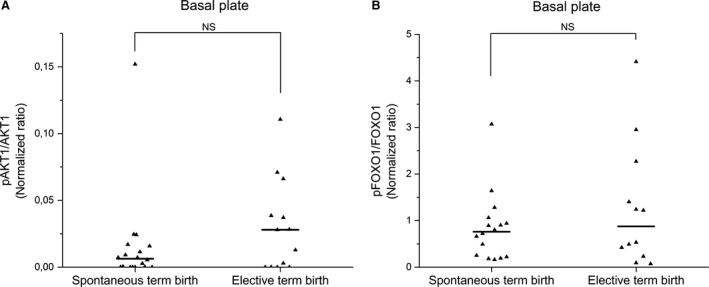
Phosphorylation of AKT1 and FOXO1 in spontaneous and elective births. The phosphorylation at Ser473 of AKT1 (pAKT1) was quantitated and compared to total AKT1 (spontaneous term birth *n *=* *18, elective term birth *n *=* *13) (**A**) and phosphorylation at Ser256 of FOXO1 (pFOXO1) was quantitated and compared to total FOXO1 (spontaneous term birth *n *=* *16, elective term birth *n *=* *12) (**B**) by Western blotting. NS means not significant change according to Mann–Whitney *U*‐test. Solid lines denote median values.

CPPED1 has been shown to directly dephosphorylate Ser473 of AKT1 in cancer tissue 19. Furthermore, a downstream target of AKT1 is forkhead box protein O1 (FOXO1, also known as forkhead homologue in rhabdomyosarcoma [FKHR]) 42, which is located in the same transcriptional complex as progesterone receptor B 43, 44 and regulates decidual transcription of certain genes 45, 46. Therefore, we investigated the phosphorylation status of AKT1 at Ser473 and FOXO1 at Ser256 in the human placenta by Western blotting to find out whether lower levels of CPPED1 during spontaneous labour are associated with increased phosphorylation of AKT1 and FOXO1. When we quantitated and compared samples from both spontaneous and elective caesarean labour, we found no detectable changes in the degree of phosphorylation at either Ser473 of AKT1 (*P *=* *0.211) or Ser256 of FOXO1 (*P *=* *0.61; Fig. 5).

**Figure 6:**
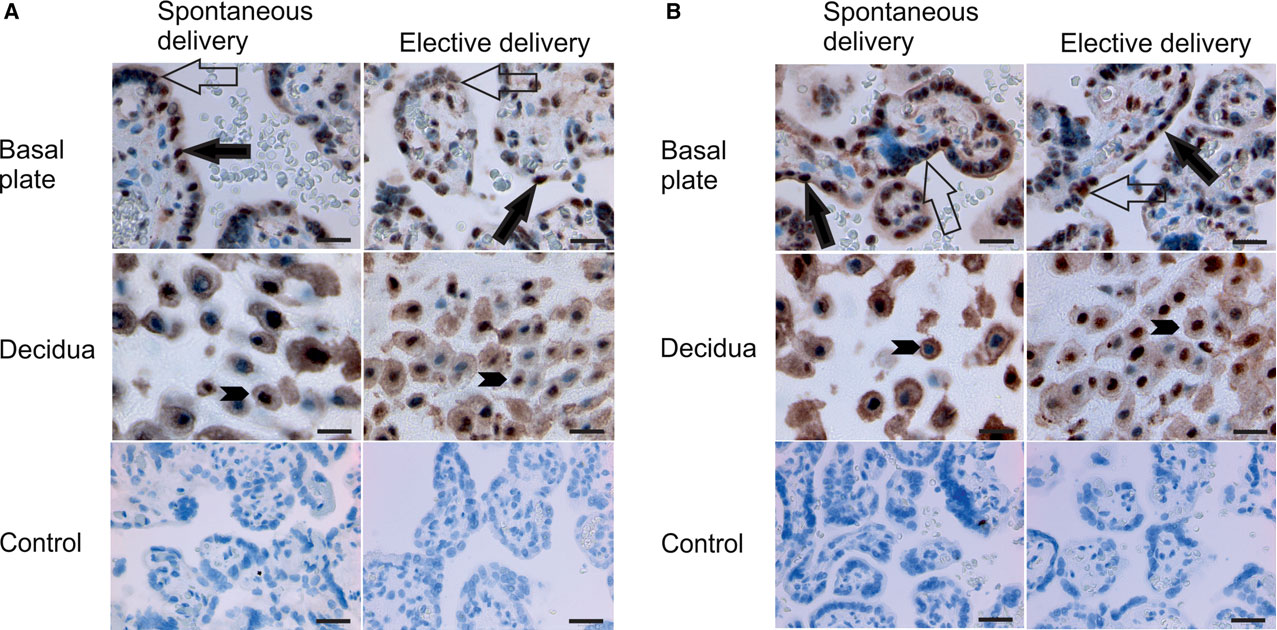
Placental localization of phosphorylated FOXO1 and phosphorylated FOXO3 in spontaneous and elective births. Immunostaining of phosphorylated FOXO1 (**A**) and phosphorylated FOXO3 (**B**) in full‐term placentas after spontaneous and elective delivery. Immunostaining is indicated by filled large arrows in cytotrophoblasts, unfilled large arrows in syncytiotrophoblasts and filled arrowheads in decidual trophoblasts. Original magnification is 40× in samples and 20× in controls. Controls represent isotype controls for immunostaining. Scale bars, 16 μm and 25 μm for samples and controls, respectively.

Previous reports have indicated that phosphorylation of FOXO1 affects its function and localization in cells 47, 48, 49 and that AKT1 is additionally able to phosphorylate FOXO3 (also known as forkhead homologue in rhabdomyosarcoma‐like 1 [FKHRL1])^50^. Therefore, we used immunohistochemistry to examine the expression of FOXO1 and FOXO3 in human placenta. We detected phosphorylated FOXO1 and phosphorylated FOXO3 in syncytio‐ and cytotrophoblasts as well as in decidual trophoblasts (Fig. 6). There was strong phosphorylated FOXO1 and FOXO3 staining in the nuclei of trophoblasts and moderate amounts in the cytosol. However, we did not detect any differences in the amount or subcellular localization of phosphorylated FOXO1 and FOXO3 when we compared placenta samples from spontaneous and elective caesarean deliveries. These results suggest that the role of CPPED1 in the timing of spontaneous birth is independent of the phosphorylation status of AKT1 and FOXO1.

### Silencing of CPPED1 affects gene expression related to inflammation and blood vessel development

To get initial insight into the function of CPPED1 in placenta, we silenced *CPPED1* post‐transcriptionally with small interfering RNA (siRNA) in HTR‐8/SVneo cells. These cells are of human origin and they have extravillous invasive trophoblast characteristic. Based on qRT‐PCR, the silencing of *CPPED1* at mRNA level was 68% (Fig. S2). When gene expression levels of HTR‐8/SVneo cells were analysed using RNA sequencing, there were 147 genes that were either up (*n *=* *77)‐ or down (*n *=* *70)‐regulated by *CPPED1* silencing compared to control cells. When the differentially expressed genes (Tables S6 and S7) were grouped according to their GO biological processes and molecular functions (Table S8), the inflammation and blood vessel and vasculature development‐related pathways were among the top affected pathways. For example, in the GO molecular functions analysis (Table S8), cytokine activity (GO:0005125) had the lowest *P*‐value (8.9 × 10^−11^) and included differentially expressed genes: *CXCL8*,* CXCL1*,* IL6*,* CCL2*,* CCL7*,* IL1*β, *GDF15*,* VSTM1*,* CXCL2*,* AREG*,* IL24*,* IL1*α, *CXCL6*,* EDN1* and *IL18* (Tables S6 and S7). Moreover, insulin‐like growth factor binding protein 5 (IGFBP5) and phosphoinositide‐3‐kinase interacting protein 1 (PIK3IP1) were significantly up‐regulated, and phosphatidylinositol‐4,5‐bisphosphate 3‐kinase catalytic subunit gamma (PIK3CG) was significantly down‐regulated in the case of lowered levels of CPPED1. These changes putatively inhibit PI3K/AKT signalling pathway.

## Discussion

Our aim in the present study was to identify placental proteins that have functional roles in the initiation of term labour. We compared the human placenta proteome after spontaneous and elective caesarean term delivery at two tissue locations, the basal and chorionic plates, to identify biochemical pathways associated with spontaneous term birth. We identified statistically significant differences in the quantity of ten proteins between spontaneous and elective caesarean term deliveries after uncomplicated pregnancies. The quantitative changes were significant in either the basal or the chorionic plates or in both.

A secondary aim of our study was to compare the proteomes of the chorionic and basal plates to identify specific protein signatures for the foetal (chorionic plate) and the maternal (basal plate) sides of the placenta and to verify spontaneous term birth‐dependent changes in different placenta locations. We identified 19 location‐dependent proteins. Among these proteins, 11 were present at higher levels in the chorionic plate. The remaining eight proteins were present at higher levels in the basal plate, suggesting that they may have a role in the placenta–uterine wall interface. To the best of our knowledge, thus far only one other proteomic study has compared placenta sides 36. Kedia *et al*. analysed uncomplicated vaginal and caesarean deliveries with particular emphasis on low molecular weight proteins and peptides. However, none of the proteins identified in our study were linked to a specific placenta region by Kedia *et al*. Furthermore, our finding that levels of these proteins differed depending on location indicates that standardized collection of tissue samples from the same placental location is essential for analyses.

One of our identified proteins was the recently characterized CPPED1 19, 41. Additionally, we detected a genetic association between the length of term pregnancy and *CPPED1* but not for any other genes encoding detected proteins. Therefore, we investigated CPPED1 and its potential function during spontaneous term birth in more detail. We found that the lower levels of CPPED1 in spontaneous term birth compared to elective caesarean term delivery were associated with a lower rate of *CPPED1* transcription. Proteomics revealed similar CPPED1 levels in the basal and chorionic plates, indicating that CPPED1 is not restricted to either the foetal or maternal side of the placenta. The reduced CPPED1 levels that we observed in association with spontaneous term birth compared to elective term birth suggest that its major function takes place until onset of labour but not during labour.

Zhuo *et al*. 19 detected decreased levels of CPPED1 mRNA in bladder cancer but not in pancreatic, liver, stomach, colon or renal cancer. They also found that CPPED1 has serine/threonine‐protein phosphatase activity and can inactivate AKT1 through specific dephosphorylation of Ser473. AKT1 is a critical component of the PI3K/AKT signalling pathway, which regulates multiple processes including metabolism, protein synthesis, cell cycle, proliferation, angiogenesis, apoptosis and survival 13, 14, 15. Downstream targets of AKT1 include FOXO1 42 and FOXO3 50, 51. FOXO1 acts as a DNA‐binding transcription factor 52, 53 and regulates cell cycle and inflammation genes 52. Phosphorylation of FOXO1 by AKT1 may cause localization to the cytoplasm and subsequent degradation of FOXO1 54, 55. In addition to regulation by phosphorylation, FOXO1 and FOXO3 are also regulated by various types of post‐translational modifications such as acetylation. Deacetylation by NAD‐dependent protein deacetylase sirtuin‐1 (SIRT1) results in the nuclear retention of FOXO1 and FOXO3 48, 56.

A previous study detected AKT1 in both villous and extravillous trophoblasts of human placenta 57. We found that not only CPPED1 but also FOXO1 and FOXO3 were expressed in cytotrophoblasts, syncytiotrophoblasts and decidual extravillous trophoblasts in human placenta. Interestingly, the majority of phosphorylated FOXO1 and FOXO3 was detected in the nuclei of these cells. Due to their nuclear localization, these phosphorylated FOXOs were presumably functionally active and deacetylated 48. In the present study, immunohistochemistry revealed no differences in subcellular localization and amounts of phosphorylated FOXO1 and FOXO3 between the placentas of spontaneous and elective caesarean term births. Nor did we observe any changes in phosphorylation levels of AKT1 and FOXO1 associated with lower CPPED1 levels. This suggests that CPPED1 does not a regulate AKT1 in AKT‐mediated signalling pathway in placenta. Interestingly, when we used siRNAs in reducing the CPPED1 levels in immortalized extravillous invading trophoblasts, we noticed up‐regulation of the negative regulators of PI3K/AKT signalling pathway upstream of AKT1. However, the most significant affected pathways involved the cytokine activity and blood vessel development. It is known, that tumour invasion and trophoblastic invasion share the same biochemical mediators, such as matrix metalloproteinases, cytokines and growth factors, and that trophoblast invasion and vascular remodelling are essential during pregnancy 58. Moreover, trophoblasts can alter the local immune environment of the decidua by allowing proinflammatory environment in early invasive phase of formation of placental vessels 59. Possibly, CPPED1 may also have a role in placentation and promote this proinflammatory effect.

In conclusion, the labour‐associated molecular mechanisms that take place in the placenta before and during spontaneous term birth are poorly understood. We have described protein signatures that were characteristic for the delivery type as well as the placental location, thereby providing important information about local and general changes in the placenta during parturition. We identified CPPED1 as a promising marker for spontaneous term delivery, confirmed a highly significant genetic association between intronic SNPs in the *CPPED1* gene and GA and identified proinflammatory pathways affected by lowered CPPED1 levels. To date, CPPED1 has been shown to have different effects depending on the cell type 19, 41. Accordingly, CPPED1 might affect differently in early and late pregnancy trophoblasts. Therefore, additional studies are required to further specify the role of CPPED1 in regulation of inflammatory mediators in trophoblasts and to identify the downstream targets of CPPED1 at the onset of spontaneous labour.

## Conflict of interest

The authors confirm that there are no conflicts of interest.

## Supporting information


**Appendix S1** Materials and methods.
**Table S1**. Protein levels and statistical significance.
**Table S2**. Protein identification.
**Table S3**. Functional classification of spontaneous birth–associated placental proteins.
**Table S4**. Analysis of SNPs within and near *ACTB, A2M, B2M, CPPED1, CYB5A, HBG2, KRT8, KRT19, PRDX2,* and *SERPINB2* for associations with gestational age in infants born at term.
**Table S5**. Means and medians of gestational age for infants with each genotype for the *CPPED1* SNPs associated with gestational age.
**Table S6**. Downregulated genes after CPPED1 silencing in HTR8/SVneo cells.
**Table S7**. Upregulated genes after *CPPED1* silencing in HTR8/SVneo cells.
**Table S8**. Functional classification of differentially expressed genes after post‐transcriptional silencing of CPPED1 in HTR8/SVneo cells.

**Figure S1** Representative 2D gel of human placenta tissue after spontaneous term birth. Placenta proteins (50 μg) collected from the basal plate of the placenta were labeled with Cy5 (minimal difference gel electrophoresis) and separated by isoelectic focusing (pH4–7, 24 cm) and SDS‐PAGE. Positions of spots that were significantly changed are indicated.

**Figure S2** The effect of siRNA on *CPPED1* mRNA levels. CPPED1 was post‐transcriptionally silenced in HTR8/SVneo cells that is a human placental trophoblast continuous cell line. RNA was isolated from *CPPED1* silenced cells and compared to RNA from control cells. In the figure, *CPPED1* expression levels are shown as determined by qRT‐PCR (A) and high throughput RNA sequencing (B). Relative mRNA levels were normalized to mRNA levels of the housekeeping gene *CYC1* (A). Reads per kilobase of exon per million reads mapped (RKPM) is a normalized gene counts value determined in the transcriptomic analysis (B). The columns show the mean value of triplicate samples; the maximum and minimum values are also indicated.
