# Supplementary material for: Expression of CPPED1 in human trophoblasts is associated with timing of term birth
Source: J Cell Mol Med. 2017 Nov 29;22(2):968–81. doi: 10.1111/jcmm.13402 (PMC5783879; doi:10.1111/jcmm.13402)
Supplement: Supplementary file 1 — Appendix S1 Materials and methods. Table S1. Protein levels and statistical significance. Table S2. Protein identification. Table S3. Functional classification of spontaneous birth–associated placental proteins. Table S4. Analysis of SNPs within and near ACTB, A2M, B2M, CPPED1, CYB5A, HBG2, KRT8, KRT19, PRDX2, and SERPINB2 for associations with gestational age in infants born at term. Table S5. Means and medians of gestational age for infants with each genotype for the CPPED1 SNPs associated with gestational age. Table S6. Downregulated genes after CPPED1 silencing in HTR8/SVneo cells. Table S7. Upregulated genes after CPPED1 silencing in HTR8/SVneo cells. Table S8. Functional classification of differentially expressed genes after post‐transcriptional silencing of CPPED1 in HTR8/SVneo cells. [file JCMM-22-968-s001.docx]

**MATERIALS AND METHODS (extended)**

**Two-dimensional minimal-difference gel electrophoresis**

After acetone precipitation, samples were resuspended in urea buffer (7 M urea, 2 M thiourea, 4% [w/v] CHAPS, and 30 mM Tris pH 8.5), sonicated, and centrifuged. We quantified protein in the supernatants with a Bradford-based assay in accordance with the manufacturer’s instructions (Roti®-Nanoquant, Carl Roth) and stored aliquots at −70°C. Protein labeling was performed with CyDye DIGE Fluor minimal dyes (GE Healthcare) in accordance with the manufacturer’s protocol, with 400 pmol dye/50 μg protein. The pooled standard was labeled with Cyanine dye (Cy) 2, and single samples were labeled with Cy3 and Cy5 by the dye-swapping approach. We separated proteins by incubating immobilized pH gradient (IPG) strips (pH 4–7, 24 cm, GE Healthcare) overnight in 650 μl of rehydration buffer (7 M urea, 2 M thiourea, 4% [w/v] CHAPS, 130 mM [w/v] DTT, 2% [v/v] carrier ampholytes 3–10, and cOmplete Mini Protease Inhibitor Cocktail [Roche]). Isoelectric focusing (IEF) after sample cup loading at the anode was carried out with the IPGphor 3 system (GE Healthcare) under paraffin oil for 80 kVh. SDS-PAGE was performed overnight on 12.5% polyacrylamide gels with the Ettan DALT II system (GE Healthcare) at 1–2 W per gel, 12°C. We used a Typhoon 9400 (GE Healthcare) to detect fluorescence signals and analyzed 2D gels with Delta2D 4.4 (Decodon).

**Mass spectrometry**

After detection of the fluorescence signals (see above) and silver staining, we matched labeled and unlabeled protein patterns with the 2D-PAGE image analysis software Melanie 3.09 (GeneBio). Spots with correctly matched centers were excised, digested with recombinant trypsin (Roche), and prepared as described previously (**Ohlmeier S, Mazur W, Salmenkivi K*, et al*.** Proteomic studies on receptor for advanced glycation end product variants in idiopathic pulmonary fibrosis and chronic obstructive pulmonary disease. *Proteomics Clin Appl.* 2010; 4: 97-105). Extracted and dried peptides were dissolved in 5 μl of α-cyano-3-hydroxycinnamic acid (98%, recrystallized from ethanol-water, 5 mg/ml in 50% acetonitrile and 0.1% trifluoroacetic acid), and 0.5 μl of the dissolved peptides was applied to the sample plate by the dried-droplet method. Peptide masses were measured with an UltrafleXtreme MALDI-TOF/TOF (Bruker). Proteins were identified according to their spot-specific peptide mass fingerprint and/or peptide sequence with the bioinformatic tool BioTools Version 3.2 (Bruker) with the following search parameters: MS tolerance, 30 ppm; MSMS tolerance, 0.7 Da; enzyme, trypsin; engine, Mascot version 2.4.0; database, NCBInr; and modifications, carbamidomethyl (Cys) and optional oxidation of methionine (up to one missed cleavage).

**Inclusion criteria for the study population in the genetic study of gestational age**

The population comprised singleton infants born at term (GA from 38 weeks + 0 days to 41 weeks + 6 days) after spontaneous onset of labor for mothers with at least two term deliveries without any major pregnancy or labor-associated complications such as fetal congenital anomalies or growth restriction, preeclampsia, polyhydramnios, placental abruptions or requirements of special care of the newborn.

**Genetic study and genotyping**

DNA was extracted from whole blood with the UltraClean DNA Blood Isolation Kit (MO BIO Laboratories) or from umbilical cord tissue with the Gentra Puregene Tissue Kit (Qiagen). DNA samples were genotyped with the Infinium HumanCoreExome BeadChip (Illumina) by the Technology Centre, Institute for Molecular Medicine Finland (FIMM), University of Helsinki, Finland. SNPs included in the genotyping chip with minor allele frequency > 0.01 located within these genes and those 20 kb up- or downstream of each gene were analyzed. SNPs that deviated from Hardy–Weinberg equilibrium and those with a genotyping rate of <0.9 were excluded. Regions surrounding *CPPED1* SNPs rs11643593 and rs8048866 were amplified from their DNA samples by PCR with primer pairs 5′-AGACAGCCCACCAAATGGGA-3′ and 5′-TCCACGTGCAAGTTTTTCACA-3′ for rs11643593 and 5′-AAGGTGATTAGCTCTGCTGGG-3′ and 5′-CTATCCTGGCAGGTAAACAAATGA-3′ for rs8048866. PCR fragments were Sanger sequenced, and we assessed associations between SNP genotypes and mRNA levels of CPPED1 by the Kruskal–Wallis test with SPSS Statistics 20.0 (IBM Corporation).

**Western blotting**

Tissue samples were sonicated and centrifuged, and protein amounts in the supernatants were determined. Proteins (20 µg) were separated by SDS-gel electrophoresis and electrotransferred onto 0.45 μm nitrocellulose membrane (Thermo Scientific) as described previously (**Karjalainen MK, Ojaniemi M, Haapalainen AM*, et al*.** CXCR3 Polymorphism and Expression Associate with Spontaneous Preterm Birth. *J Immunol.* 2015; 195: 2187-98.). Membranes were incubated overnight in blocking solution (3% bovine serum albumin in PBS [Thermo Fisher Scientific], 4°C).

**Immunohistochemistry**

We collected placenta samples from the chorionic and basal plates as described previously (**Karjalainen MK, Ojaniemi M, Haapalainen AM*, et al*.** CXCR3 Polymorphism and Expression Associate with Spontaneous Preterm Birth. *J Immunol.* 2015; 195: 2187-98.). We chose 12 placentas from full-term uncomplicated pregnancies for the analyses, six from deliveries by spontaneous onset of labor with vaginal delivery and six from elective caesarean deliveries due to inadequate diameter of maternal pelvis prior to onset of active labor. Samples were embedded in paraffin and cut in 4 µm slices, deparaffinized, and dehydrated. For antigen retrieval, Tris-EDTA buffer was used for the samples. Endogenous peroxidase activity was blocked with blocking solution (S2023; DAKO).

**Quantitative PCR**

Primers and probes were forward 5′-ACCCAAATTCTTCGTTCTGTG-3′ and reverse 5′-CAAGGACCAGTGGGATGG-3′ for *CPPED1* (with UPL probe 86) and forward 5′-ATAAAGCGGCACAAGTGGTCA-3′ and reverse 5′-GATGGCTCTTGGGCTTGAGG-3′ for *CYC1* (with UPL probe 47). For relative quantification, mRNA levels were normalized against *CYC1* mRNA according to the ΔΔC_t_ method. *CYC1* was chosen as a reference gene based on previous reports (**Cleal JK, Day P, Hanson MA, Lewis RM.** Measurement of housekeeping genes in human placenta. *Placenta.* 2009; 30: 1002-3. **Cleal JK, Day PL, Hanson MA, Lewis RM.** Sex differences in the mRNA levels of housekeeping genes in human placenta. *Placenta.* 2010; 31: 556-7. **Drewlo S, Levytska K, Kingdom J.** Revisiting the housekeeping genes of human placental development and insufficiency syndromes. *Placenta.* 2012; 33: 952-4.). All samples were measured as triplicates. The identities of qPCR-amplified products were further confirmed by agarose gel electrophoresis (size of the PCR product) and DNA sequencing (sequence of the PCR product). Statistical analyses were conducted with SPSS Statistics 20.0 (IBM Corporation). Significant differences in expression levels were identified with the nonparametric Mann–Whitney *U* test.

**Transfection with small interfering RNA**

The HTR-8/SVneo (CRL-3271) trophoblast cell line was purchased from ATCC, and the cells were cultured in RPMI 1640 medium (Invitrogen Life Technologies) supplemented with 10 % fetal bovine serum (Invitrogen Life Technologies), 100 U/ml penicillin, 100 mg/ml streptomycin, 2 mM glutamine and 1 mM sodium pyruvate. Subculturing was performed with 0.05 % trypsin / 0.02 % EDTA.

On the day of transfection, 100 nM of pooled siRNA (pairs 1-3) Lipofectamine 3000 (Invitrogen Life Technologies) complexes were prepared in serum free media, after which the complexes were added into approximately 0.1 × 10^6^ cells in suspension. Cells were then seeded in 12-well cell culture plates and incubated for 24 h (= reverse transfection). The 30 nM of siRNA Universal Negative Control was used as a negative control. The next day, media was removed from wells and 1 ml of complete media was added. CPPED1 and negative control siRNA Lipofectamine 3000 transfection complexes were prepared in serum free media as mentioned earlier and added to cells. After 48 hours of an additional incubation (= forward transfection), cells were harvested and RNA isolated by using Trizol reagent (Sigma) and RNeasy Micro Kit (Qiagen).

The specific siRNA duplexes (siRNA pairs 1-3) targeted against human CPPED1 were purchased from Ambion Dharmacon. The pairs were: CAAACUACACCUGAUUGUA[dT][dT] UACAAUCAGGUGUAGUUUG[dT][dT] (pair1), GUCAAAGUCUGCCUAUCGA[dT][dT] UCGAUAGGCAGACUUUGAC[dT][dT] (pair2), CCUAUCGACCUCAAUUCCA[dT][dT] UGGAAUUGAGGUCGAUAGG[dT][dT] (pair 3). The negative control was siRNA Universal Negative Control #1 (Sigma).

**Supplementary Table S1. Protein levels and statistical significance.** Spot numbers and identified proteins according to Figure S1 and Table 1 are shown. Protein levels are represented by mean normalized spot volumes for elective (E) and spontaneous (S) term birth in samples collected from the basal (Ba) or chorionic (Ch) plates of the placenta. Statistical significance according to Student’s *t*-test with *p* < 0.05 as the cut-off is indicated for comparisons E vs S and Ba vs Ch in the corresponding subgroups Ba and Ch or E and S. Only the amount of one protein (ACTB) changed significantly in both comparisons (E vs S and Ba vs Ch).

| **Spot** | **Protein** | **UniProt-KB** | **Description** | **Mean normalized volumes** | | | | **t-Test** | | | |
| --- | --- | --- | --- | --- | --- | --- | --- | --- | --- | --- | --- |
|  |  |  |  | **Ba** | | **Ch** | | **E vs S** | | **Ba vs Ch** | |
|  |  |  |  | **E** | **S** | **E** | **S** | **Ba** | **Ch** | **E** | **S** |
| **Elective (E) vs spontaneous (S) birth** | | | | | | | | | | | |
| 46* | ACTB | P60709 | Actin, cytoplasmic 1 (fragment) | 110 | 174 | 238 | 269 | 0,049 | 0,721 | 0,007 | 0,271 |
| 1 | A2M | P01023 | α-2-macroglobulin | 343 | 214 | 385 | 261 | 0,024 | 0,092 | 0,581 | 0,177 |
| 2 |  |  |  | 338 | 208 | 371 | 234 | 0,023 | 0,003 | 0,527 | 0,427 |
| 3 |  |  |  | 347 | 223 | 335 | 202 | 0,029 | 0,004 | 0,801 | 0,623 |
| 49 | B2M | P61769 | β-2-microglobulin | 386 | 434 | 267 | 419 | 0,574 | 0,021 | 0,065 | 0,860 |
| 39 | CPPED1 | Q9BRF8 | Serine/threonine-protein phosphatase (isoform 1) | 312 | 183 | 275 | 153 | 0,029 | 0,043 | 0,310 | 0,654 |
| 44 | CYB5A | P00167 | Cytochrome b5 (isoform 1 or 2) | 327 | 187 | 351 | 220 | 0,027 | 0,003 | 0,685 | 0,299 |
| 47 | HBG2 | P69892 | Hemoglobin subunit γ-2 | 330 | 127 | 341 | 150 | 0,023 | 0,002 | 0,896 | 0,375 |
| 48 |  |  |  | 400 | 169 | 451 | 220 | 0,009 | 0,009 | 0,549 | 0,091 |
| 13 | KRT8 | P05787 | Keratin, type II cytoskeletal 8 (isoform 1 or 2) | 246 | 283 | 217 | 368 | 0,441 | 0,002 | 0,514 | 0,088 |
| 14 |  |  | isoform 1 or 2 | 254 | 272 | 197 | 338 | 0,730 | 0,015 | 0,311 | 0,238 |
| 15 |  |  | isoform 1 or 2 | 191 | 293 | 209 | 427 | 0,318 | 0,015 | 0,695 | 0,279 |
| 30* |  |  | fragment of isoform 1 or 2 | 173 | 315 | 275 | 658 | 0,459 | 0,019 | 0,235 | 0,142 |
| 31* |  |  | fragment of isoform 1 or 2 | 165 | 308 | 176 | 481 | 0,452 | 0,016 | 0,871 | 0,414 |
| 21 | KRT19 | P08727 | Keratin, type I cytoskeletal 19 | 266 | 246 | 232 | 365 | 0,806 | 0,032 | 0,676 | 0,117 |
| 29* |  |  | C-terminal KRT19 fragment | 232 | 278 | 260 | 428 | 0,580 | 0,031 | 0,579 | 0,128 |
| 42 | PRDX2 | P32119 | Peroxiredoxin-2 (isoform 1) | 317 | 191 | 349 | 232 | 0,008 | 0,111 | 0,646 | 0,282 |
| 22 | SERPINB2 | P05120 | Plasminogen activator inhibitor 2 | 239 | 209 | 203 | 105 | 0,489 | 0,039 | 0,303 | 0,054 |
| **Basal plate (Ba) vs chorionic plate (Ch)** | | | | | | | | | | | |
| 46* | ACTB | P60709 | Actin, cytoplasmic 1 (fragment) | 110 | 174 | 238 | 269 | 0,049 | 0,721 | 0,007 | 0,271 |
| 38 | ANXA3 | P12429 | Annexin A3 | 336 | 251 | 511 | 709 | 0,391 | 0,223 | 0,236 | 0,003 |
| 45* | ANXA5 | P08758 | Annexin A5 (fragment) | 143 | 198 | 223 | 213 | 0,073 | 0,877 | 0,023 | 0,785 |
| 41 | APCS | P02743 | Serum amyloid P-component | 257 | 290 | 485 | 799 | 0,528 | 0,059 | 0,067 | 0,001 |
| 32 | CLU | P10909 | Clusterin (isoform 3 or fragments of isoforms 1,2,4, 5) | 241 | 274 | 476 | 784 | 0,674 | 0,079 | 0,065 | 0,003 |
| 33 |  |  | isoform 3 or fragments of isoforms 1,2,4, 5 | 251 | 282 | 541 | 839 | 0,685 | 0,106 | 0,038 | 0,003 |
| 34 |  |  | isoform 3 or fragments of isoforms 1,2,4, 5 | 314 | 284 | 764 | 1051 | 0,705 | 0,205 | 0,013 | 0,001 |
| 5 | EEF2 | P13639 | Elongation factor 2 | 208 | 202 | 155 | 87 | 0,944 | 0,349 | 0,559 | 0,028 |
| 23* | FGB | P02675 | Fibrinogen β (C-terminal fragment in D dimer) | 257 | 368 | 578 | 902 | 0,181 | 0,202 | 0,019 | 0,039 |
| 24* |  |  | C-terminal fragment in D dimer | 261 | 331 | 648 | 1050 | 0,316 | 0,192 | 0,026 | 0,020 |
| 25* |  |  | C-terminal fragment in D dimer | 273 | 315 | 691 | 1076 | 0,604 | 0,226 | 0,039 | 0,014 |
| 26* |  |  | C-terminal fragment in D dimer | 243 | 376 | 422 | 681 | 0,093 | 0,117 | 0,031 | 0,070 |
| 6 | FGG | P02679 | Fibrinogen gamma (isoform γ-A or –B) | 150 | 381 | 311 | 519 | 0,176 | 0,138 | 0,049 | 0,492 |
| 7 |  |  | isoform γ-A or –B | 165 | 359 | 354 | 550 | 0,165 | 0,188 | 0,044 | 0,293 |
| 16 |  |  | isoform γ-A or –B | 275 | 243 | 434 | 499 | 0,559 | 0,426 | 0,022 | 0,009 |
| 43 | FTL | P02792 | Ferritin light chain | 427 | 407 | 217 | 148 | 0,929 | 0,268 | 0,177 | 0,001 |
| 4 | GSN | P06396 | Gelsolin (isoform 1) | 444 | 279 | 533 | 724 | 0,079 | 0,111 | 0,398 | 0,001 |
| 40 | HIBADH | P31937 | 3-hydroxyisobutyrate dehydrogenase | 293 | 292 | 255 | 178 | 0,965 | 0,139 | 0,399 | 0,048 |
| 11 | HNRNPK | P61978 | Heterogeneous nuclear ribonucleoprotein K (isoform 1, 2 or 3) | 217 | 272 | 197 | 111 | 0,158 | 0,138 | 0,679 | 0,004 |
| 27 | HSD17B1 | P14061 | Estradiol 17-β-dehydrogenase 1 | 254 | 282 | 203 | 144 | 0,591 | 0,097 | 0,263 | 0,011 |
| 28 |  |  |  | 277 | 300 | 209 | 147 | 0,664 | 0,115 | 0,189 | 0,009 |
| 10 | LMNB1 | P20700 | Lamin-B1 | 254 | 252 | 224 | 165 | 0,964 | 0,157 | 0,495 | 0,032 |
| 17 | LUM | P51884 | Lumican | 287 | 222 | 804 | 1112 | 0,672 | 0,208 | 0,043 | 0,002 |
| 18 |  |  |  | 293 | 226 | 695 | 1021 | 0,626 | 0,211 | 0,058 | 0,005 |
| 19 |  |  |  | 277 | 207 | 735 | 1571 | 0,659 | 0,122 | 0,064 | 0,018 |
| 20 |  |  |  | 222 | 220 | 501 | 1152 | 0,978 | 0,111 | 0,054 | 0,029 |
| 36 | TPM1 | P09493 | Tropomyosin α-1 chain (isoform 8) | 324 | 304 | 681 | 920 | 0,829 | 0,362 | 0,025 | 0,024 |
| 37 |  |  | isoform 1 or 9 | 324 | 294 | 650 | 854 | 0,777 | 0,365 | 0,055 | 0,014 |
| 35 | TPM2 | P07951 | Tropomyosinβ chain (isoform 2) | 339 | 325 | 831 | 1272 | 0,910 | 0,296 | 0,026 | 0,031 |
| 8 | VPS35 | Q96QK1 | Vacuolar protein sorting-associated protein 35 | 275 | 274 | 172 | 91 | 0,931 | 0,084 | 0,061 | 0,004 |
| 9 |  |  |  | 246 | 253 | 179 | 116 | 0,923 | 0,268 | 0,214 | 0,025 |
| 12 | WARS | P23381 | Tryptophan-tRNA ligase (isoform 1 or 2) | 258 | 283 | 182 | 124 | 0,619 | 0,285 | 0,149 | 0,008 |

**Supplementary Table S2. Protein identification.** Parameters of protein identification, including MS and MSMS Mascot scores as well as sequence coverage (SC) and covered peptides (P). The total number of amino acids and the sequence covered by MS are also indicated. If the identified spot allowed for the presence of several isoforms, amino acids and sequence coverage are indicated for the most common isoform 1 or A. If both spot position and sequence coverage indicate the presence of a protein fragment, it is marked by an asterisk.

| **Spot** | **Protein** | **UniProt-KB** | **Description** | **Amino acids** | **Amino acids covered by MS** | **Score**  **(MS, MSMS)** | **SC (P)**  **(MS, MSMS)** |
| --- | --- | --- | --- | --- | --- | --- | --- |
| 1 | A2M | P01023 | α-2-macroglobulin | 1474 | 215-732 | -, 148.4 | -, 3.8(4) |
| 2 | A2M | P01023 | α-2-macroglobulin | 1474 | 215-945 | 96.6, 31.0 | 8.8(9), 0.7(1) |
| 3 | A2M | P01023 | α-2-macroglobulin | 1474 | 320-732 | 65.4, 119.6 | 8.2(9), 21.9(3) |
| 4 | GSN | P06396 | Gelsolin (isoform 1) | 755 | 33-740 | 227.0, 73.0 | 23.8(16), 1.4(1) |
| 5 | EEF2 | P13639 | Elongation factor 2 | 858 | 367-801 | 117.0, 42.0 | 23.8(11), 3.1(1) |
| 6 | FGG | P02679 | Fibrinogen γ (isoform γ-A or –B) | 453^A^ | 89-417^A^ | 597.0, 113.0 | 60.7(18), 16.8(6) |
| 7 | FGG | P02679 | Fibrinogen γ (isoform γ-A or –B) | 453^A^ | 32-417^A^ | 559.0, 121.0 | 49.0(15), 14.6(5) |
| 8 | VPS35 | Q96QK1 | Vacuolar protein sorting-associated protein 35 | 796 | 45-778 | 78.5,- | 17.7(13),- |
| 9 | VPS35 | Q96QK1 | Vacuolar protein sorting-associated protein 35 | 796 | 15-768 | 142.0, 92.0 | 12.9(8), 1.9(1) |
| 10 | LMNB1 | P20700 | Lamin-B1 | 586 | 15-577 | 494.0, 127.0 | 57.0(33), 17.4(6) |
| 11 | HNRNPK | P61978 | Heterogeneous nuclear ribonucleoprotein K (isoform 1, 2 or 3) | 463^1^ | 36-316^1^ | 52.6, 39.0 | 8.4(3), 2.4(1) |
| 12 | WARS | P23381 | Tryptophan-tRNA ligase (isoform 1 or 2) | 471^1^ | 82-471^1^ | 499.0, 85.0 | 68.7(25), 22.8(7) |
| 13 | KRT8 | P05787 | Keratin, type II cytoskeletal 8 (isoform 1 or 2) | 483^1^ | 24-414^1^ | 561.0, 99.0 | 50.2(28), 16.5(6) |
| 14 | KRT8 | P05787 | Keratin, type II cytoskeletal 8 (isoform 1 or 2) | 483^1^ | 24-454^1^ | 845.0, 121.0 | 60.2 (32), 18.8(7) |
| 15 | KRT8 | P05787 | Keratin, type II cytoskeletal 8 (isoform 1 or 2 ) | 483^1^ | 24-414^1^ | 181.0, 57.0 | 38.5(17), 2.3(1) |
| 16 | FGG | P02679 | Fibrinogen γ (isoform γ-A or –B) | 453 | 32-432 | 585.0, 88.0 | 60.7(19), 20.5(7) |
| 17 | LUM | P51884 | Lumican | 338 | 70-338 | 207.0, 53.0 | 21.0(8), 1.9(3) |
| 18 | LUM | P51884 | Lumican | 338 | 70-338 | 206.0, 88.0 | 27.2(9), 6.5(2) |
| 19 | LUM | P51884 | Lumican | 338 | 70-338 | 229.0, 107.0 | 24.3(9), 6.5(2) |
| 20 | LUM | P51884 | Lumican | 338 | 70-338 | 151.0, 36.0 | 18.0(7), 10.9(3) |
| 21 | KRT19 | P08727 | Keratin, type I cytoskeletal 19 | 400 | 8-381 | 809.0, 139.0 | 65.5(27), 18.0(6) |
| 22 | SERPINB2 | P05120 | Plasminogen activator inhibitor 2 | 415 | 2-411 | 443.0, 134.0 | 43.4(18), 17.8(4) |
| 23* | FGB | P02675 | Fibrinogen β (C-terminal fragment in D dimer) | 491 | 179-491 | 725.0, 180.0 | 57.8(16), 21.1(6) |
| 24* | FGB | P02675 | Fibrinogen β (C-terminal fragment in D dimer) | 491 | 179-491 | 594.0, 104.0 | 56.4(17), 25.3(6) |
| 25* | FGB | P02675 | Fibrinogen β (C-terminal fragment in D dimer) | 491 | 179-491 | 1060.0, 162.0 | 75.0(27), 28.0(8) |
| 26* | FGB | P02675 | Fibrinogen β (C-terminal fragment in D dimer) | 491 | 164-491 | 222.0, 76.0 | 54.3(15), 6.4(1) |
| 27 | HSD17B1 | P14061 | Estradiol 17-β-dehydrogenase 1 | 328 | 4-312 | 671.0, 149.0 | 49.2(16), 30.6(7) |
| 28 | HSD17B1 | P14061 | Estradiol 17-β-dehydrogenase 1 | 328 | 23-328 | 682.0, 118.0 | 64.6(22), 29.0(6) |
| 29* | KRT19 | P08727 | Keratin, type I cytoskeletal 19 (C-terminal fragment) | 400 | 91-398 | 590.0, 91.0 | 56.0(29), 21.3(9) |
| 30* | KRT8 | P05787 | Keratin, type II cytoskeletal 8 (fragment of isoform 1 or 2) | 483 | 123-401 | 748.0, 117.0 | 66.8(30), 19.5(6) |
| 31* | KRT8 | P05787 | Keratin, type II cytoskeletal 8 (fragment of isoform 1 or 2) | 483 | 123-414 | 544.0, 96.0 | 56.8(26), 14.1(5) |
| 32 | CLU | P10909 | Clusterin (isoform 3 or fragments of isoforms 1,2,4 or 5) | 449^1^ | 183-444^1^ | 433.0, 156.0 | 36.5(10), 18.6(3) |
| 33 | CLU | P10909 | Clusterin (isoform 3 or fragments of isoforms 1,2,4 or 5) | 449^1^ | 183-444^1^ | 209.0, 209.0 | 31.4(7),10.2(2) |
| 34 | CLU | P10909 | Clusterin (isoform 3 or fragments of isoforms 1,2,4 or 5) | 449^1^ | 183-444^1^ | 231.0, 86.0 | 31.8(8), 12.4(2) |
| 35 | TPM2 | P07951 | Tropomyosin β chain (isoform 2) | 284 | 13-284 | 618.0, 77.0 | 63.0(29), 11.3(5) |
| 36 | TPM1 | P09493 | Tropomyosin α-1 chain (isoform 8) | 284 | 38-244 | 320.0, 69.0 | 44.0(18), 15.8(4) |
| 37 | TPM1 | P09493 | Tropomyosin α-1 chain (isoform 1 or 9) | 284^1^ | 36-264^1^ | 273.0, 56.0 | 47.0(19), 9.5(2) |
| 38 | ANXA3 | P12429 | Annexin A3 | 323 | 14-317 | 540.0, 108.0 | 41.2(13), 23.5(6) |
| 39 | CPPED1 | Q9BRF8 | Serine/threonine-protein phosphatase (isoform 1) | 314 | 17-312 | 326.0, 94.0 | 39.5(10), 17.2(4) |
| 40 | HIBADH | P31937 | 3-hydroxyisobutyrate dehydrogenase | 336 | 40-336 | 152.0, 69.0 | 25.6(5), 9.5(2) |
| 41 | APCS | P02743 | Serum amyloid P-component | 223 | 27-165 | 466.0, 134.0 | 34.8(11), 23.5(4) |
| 42 | PRDX2 | P32119 | Peroxiredoxin-2 (isoform 1) | 198 | 8-191 | 630.0, 159.0 | 77.2(17), 23.9(4) |
| 43 | FTL | P02792 | Ferritin light chain | 175 | 54-169 | 300.0, 134.0 | 62.9(9), 17.5(2) |
| 44 | CYB5A | P00167 | Cytochrome b5 (isoform 1 or 2) | 134^1^ | 40-91^1^ | 118.0, 67.0 | 55.2(3), 14.9(1) |
| 45* | ANXA5 | P08758 | Annexin A5 (fragment) | 320 | 7-286 | 276.0, 112.0 | 39.2(11), 11.6(3) |
| 46* | ACTB | P60709 | Actin, cytoplasmic 1 (fragment) | 375 | 19-113 | 147.0, 83.0 | 45.7(6), 17.7(2) |
| 47 | HBG2 | P69892 | Hemoglobin subunit γ-2 | 147 | 2-147 | 598.0, 148.0 | 84.2(13), 45.9(5) |
| 48 | HBG2 | P69892 | Hemoglobin subunit γ-2 | 147 | 2-147 | 556.0, 155.0 | 84.9(14), 41.8(5) |
| 49 | B2M | P61769 | β-2-microglobulin | 119 | 27-111 | 93.4, 65.9 | 61.7(6), 46.4(4) |

**Supplementary Table S3. Functional classification of spontaneous birth–associated placental proteins.** Clustering was performed by DAVID analysis according to Gene Ontology (GO) biological processes and molecular functions.

| **Category** | **GO Term** | **Genes** | **p value** | **Benjamini-Hochberg corrected p value** |
| --- | --- | --- | --- | --- |
| **Biological process** | | | | |
| GOTERM_BP_FAT | GO:0010035: response to inorganic substance | *ACTB, A2M, PRDX2* | 6.0 × 10^-3^ | 7.1 × 10^-1^ |
| GOTERM_BP_FAT | GO:0010033: response to organic substance | *A2M, B2M, KRT19, PRDX2* | 6.9 × 10^-3^ | 5.0 × 10^-1^ |
| GOTERM_BP_FAT | GO:0009611: response to wounding | *A2M, PRDX2, SERPINB2* | 3.7 × 10^-2^ | 9.2 × 10^-1^ |
| GOTERM_BP_FAT | GO:0002683: negative regulation of immune system process | *A2M, PRDX2* | 4.8 × 10^-2^ | 9.2 × 10^-1^ |
| GOTERM_BP_FAT | GO:0002237: response to molecule of bacterial origin | *B2M, PRDX2* | 5.0 × 10^-2^ | 8.7 × 10^-1^ |
| GOTERM_BP_FAT | GO:0002526:  acute inflammatory response | *A2M, PRDX2* | 5.7 × 10^-2^ | 8.6 × 10^-1^ |
| GOTERM_BP_FAT | GO:0002697: regulation of immune effector process | *A2M, B2M* | 5.8 × 10^-2^ | 8.2 × 10^-1^ |
| **Molecular function** | | | | |
| GOTERM_MF_FAT | GO:0005200: structural constituent of cytoskeleton | *ACTB, KRT19* | 4.5 × 10^-2^ | 9.2 × 10^-1^ |
| GOTERM_MF_FAT | GO:0005198: structural molecule activity | *ACTB, KRT19, KRT8* | 5.5 × 10^-2^ | 7.9 × 10^-1^ |
| GOTERM_MF_FAT | GO:0004867:  serine-type endopeptidase inhibitor activity | *A2M, SERPINB2* | 5.5 × 10^-2^ | 6.5 × 10^-1^ |
| GOTERM_MF_FAT | GO:0020037:  heme binding | *CYB5A, HBG2* | 7.2 × 10^-2^ | 6.4 × 10^-1^ |
| GOTERM_MF_FAT | GO:0046906: tetrapyrrole binding | *CYB5A, HBG2* | 7.7 × 10^-2^ | 5.8 × 10^-1^ |
| GOTERM_MF_FAT | GO:0004866: endopeptidase inhibitor activity | *A2M, SERPIN2B* | 8.6 × 10^-2^ | 5.6 × 10^-1^ |
| GOTERM_MF_FAT | GO:0030414: peptidase inhibitor activity | *A2M, SERPIN2B* | 9.1 × 10^-2^ | 5.3 × 10^-1^ |

**Supplementary Table S4. Analysis of SNPs within and near *ACTB, A2M, B2M, CPPED1, CYB5A, HBG2, KRT8*, *KRT19*, *PRDX2,* and *SERPINB2* for associations with gestational age in infants born at term.** SNPs with minor allele frequency > 0.01 that were genotyped with the HumanCoreExome chip and located 20 kb up- or downstream of each gene were analyzed in 342 infants. SNPs significant at the Bonferroni-corrected level (*p* < 6.5 × 10^−4^) are highlighted in bold.

| **Gene** | **Specific location^1^** | **Chro-mo-**  **some** | **Position^2^** | **SNP** | **Beta coefficient^3^** | ***p* value** |
| --- | --- | --- | --- | --- | --- | --- |
| *ACTB* | Downstream | 7 | 5,562,740 | rs852432 | -0.089 | 0.222 |
| *ACTB* | Upstream | 7 | 5,571,625 | rs2966449 | -0.010 | 0.155 |
| *ACTB* | Upstream | 7 | 5,582,362 | rs6967973 | 0.028 | 0.738 |
| *A2M* | Downstream | 12 | 9,205,030 | rs969936 | -0.054 | 0.589 |
| *A2M* | Downstream | 12 | 9,219,900 | rs3026229 | 0.241 | 0.030 |
| *A2M* | Intronic | 12 | 9,220,729 | rs7955940 | -0.059 | 0.409 |
| *A2M* | Intronic | 12 | 9,228,255 | rs1805659 | -0.055 | 0.448 |
| *A2M* | Exonic (Ala1103Thr) | 12 | 9,229,986 | rs113563450 | -0.150 | 0.742 |
| *A2M* | Exonic (Ile1000Val) | 12 | 9,232,268 | rs669 | -0.059 | 0.409 |
| *A2M* | Intronic | 12 | 9,250,601 | rs4882978 | 0.245 | 0.026 |
| *A2M* | Intronic | 12 | 9,267,559 | rs226381 | 0.041 | 0.553 |
| *A2M* | Upstream | 12 | 9,279,598 | rs226372 | -0.020 | 0.775 |
| *B2M* | Downstream | 15 | 45,028,710 | rs2470908 | -0.044 | 0.802 |
| *CPPED1* | Downstream | 16 | 12,737,141 | rs1651014 | -0.069 | 0.346 |
| *CPPED1* | Downstream | 16 | 12,742,340 | rs1612572 | -0.137 | 0.080 |
| *CPPED1* | 3’ untranslated region | 16 | 12,756,649 | rs9926821 | 0.169 | 0.336 |
| *CPPED1* | Exonic (Lys241Arg) | 16 | 12,758,966 | rs1713480 | 0.169 | 0.336 |
| *CPPED1* | Intronic | 16 | 12,768,284 | rs1713471 | 0.099 | 0.457 |
| *CPPED1* | Intronic | 16 | 12,784,013 | rs8045278 | -0.042 | 0.688 |
| ***CPPED1*** | **Intronic** | **16** | **12,808,242** | **rs11643593** | **0.259** | **2.7 × 10^-4^** |
| *CPPED1* | Intronic | 16 | 12,817,240 | rs882908 | -0.268 | 7.0 × 10^-4^ |
| *CPPED1* | Intronic | 16 | 12,829,006 | rs11075148 | -0.063 | 0.470 |
| ***CPPED1*** | **Intronic** | **16** | **12,846,807** | **rs8048866** | **-0.282** | **3.3 × 10^-4^** |
| *CPPED1* | Intronic | 16 | 12,850,671 | rs2865625 | -0.043 | 0.588 |
| *CPPED1* | Intronic | 16 | 12,862,007 | rs4780476 | -0.033 | 0.670 |
| *CPPED1* | Intronic | 16 | 12,863,834 | rs12921027 | -0.099 | 0.198 |
| *CPPED1* | Intronic | 16 | 12,885,808 | rs8048492 | 0.147 | 0.106 |
| *CPPED1* | Intronic | 16 | 12,894,856 | rs12598821 | 0.158 | 0.085 |
| *CPPED1* | Upstream | 16 | 12,908,309 | rs16960741 | -0.012 | 0.949 |
| *HBG2* | Downstream | 11 | 5,258,989 | rs4910736 | 0.008 | 0.903 |
| *HBG2* | Downstream | 11 | 5,268,797 | rs10488676 | 0.0006 | 0.993 |
| *HBG2* | Downstream (within *HBG1*) | 11 | 5,271,671 | rs2855039 | 0.069 | 0.395 |
| *HBG2* | Intronic | 11 | 5,275,343 | rs11036476 | -0.011 | 0.878 |
| *HBG2* | Upstream | 11 | 5,279,153 | rs5010979 | 0.008 | 0.917 |
| *KRT8* | Downstream | 12 | 53,27,3745 | rs902771 | -0.083 | 0.269 |
| *KRT8* | Downstream | 12 | 53,278,959 | rs2682332 | 0.089 | 0.438 |
| *KRT8* | Downstream | 12 | 53,283,198 | rs1907671 | 0.018 | 0.784 |
| *KRT8* | Downstream | 12 | 53,285,086 | rs11170319 | 0.034 | 0.613 |
| *KRT8* | Intronic | 12 | 53,297,320 | rs7964223 | 0.042 | 0.588 |
| *KRT8* | Intronic | 12 | 53,298,835 | rs2070910 | 0.025 | 0.7497 |
| *KRT8* | Intronic | 12 | 53,303,331 | rs17120257 | 0.025 | 0.790 |
| *KRT8* | Intronic | 12 | 53,310,411 | rs2682295 | -0.073 | 0.300 |
| *KRT8* | Intronic | 12 | 53,315,157 | rs10219651 | -0.003 | 0.977 |
| *KRT8* | Intronic | 12 | 53,335,563 | rs11170341 | 0.049 | 0.533 |
| *KRT8* | Upstream (within *KRT18*) | 12 | 53,344,830 | rs2070876 | -0.015 | 0.857 |
| *KRT8* | Upstream | 12 | 53,354,622 | rs2363635 | 0.094 | 0.255 |
| *KRT19* | Downstream (within *KRT15*) | 17 | 39,661,689 | rs8182306 | 0.208 | 0.278 |
| *KRT19* | Downstream (within *KRT15*) | 17 | 39,671,724 | rs2305556 | 0.053 | 0.548 |
| *KRT19* | Downstream (within *KRT15*) | 17 | 39,674,641 | rs1050784 | 0.006 | 0.932 |
| *KRT19* | Exonic (Glu261Lys) | 17 | 39,680,672 | rs148803515 | -0.081 | 0.712 |
| *KRT19* | Upstream | 17 | 39,686,057 | rs11650388 | 0.006 | 0.932 |
| *CYB5A* | Downstream | 18 | 71,907,776 | rs1024908 | 0.104 | 0.145 |
| *CYB5A* | Downstream | 18 | 71,915,587 | rs1788571 | 0.047 | 0.522 |
| *CYB5A* | Downstream | 18 | 71,920,369 | rs1790870 | -0.030 | 0.910 |
| *CYB5A* | Downstream | 18 | 71,920,413 | rs17804159 | 0.080 | 0.439 |
| *CYB5A* | Intronic | 18 | 71,927,586 | rs1788625 | -0.030 | 0.910 |
| *CYB5A* | Exonic (Pro96Pro) | 18 | 71,928,150 | rs7238987 | 0.011 | 0.905 |
| *CYB5A* | Intronic | 18 | 71,929,265 | rs12965502 | 0.086 | 0.244 |
| *CYB5A* | Intronic | 18 | 71,955,979 | rs1573486 | 0.070 | 0.323 |
| *CYB5A* | Upstream | 18 | 71,960,136 | rs3813106 | 0.101 | 0.279 |
| *CYB5A* | Upstream | 18 | 71,971,089 | rs1582404 | -0.012 | 0.868 |
| *CYB5A* | Upstream | 18 | 71,978,076 | rs4328535 | 0.038 | 0.570 |
| *PRDX2* | Downstream (within *MIR5684*) | 19 | 12,897,816 | rs7250961 | 0.441 | 0.014 |
| *PRDX2* | Intronic | 19 | 12,911,425 | rs10421102 | -0.244 | 0.266 |
| *PRDX2* | Upstream (within *RNASEH2A*) | 19 | 12,921,186 | rs7247284 | -0.288 | 0.177 |
| *SERBINB2* | Upstream | 18 | 61,541,617 | rs9957643 | -0.099 | 0.252 |
| *SERBINB2* | Intronic | 18 | 61,557,670 | rs1025196 | -0.102 | 0.237 |
| *SERBINB2* | Intronic | 18 | 61,559,711 | rs9630856 | -0.102 | 0.237 |
| *SERBINB2* | Exonic (Pro393Pro) | 18 | 61,570,470 | rs6102 | -0.082 | 0.354 |
| *SERBINB2* | Exonic (Ser413Cys) within SERPINB2 | 18 | 61,570,529 | rs6104 | -0.102 | 0.237 |
| *SERBINB2* | Downstream (within SERPINB10) | 18 | 61,579,377 | rs9966202 | -0.093 | 0.297 |
| *SERBINB2* | Downstream (within SERPINB10) | 18 | 61,582,751 | ra17072097 | -0.093 | 0.297 |
| *SERBINB2* | Downstream (within SERPINB10) | 18 | 61,582,867 | rs8097425 | -0.113 | 0.198 |
| *SERBINB2* | Downstream (within SERPINB10) | 18 | 61,584,726 | rs760775285 | 0.392 | 0.178 |
| *SERBINB2* | Downstream (within SERPINB10) | 18 | 61,585,260 | rs724558 | -0.093 | 0.297 |
| *SERBINB2* | Downstream (within SERPINB10) | 18 | 61,587,053 | rs17072146 | -0.212 | 0.423 |
| *SERBINB2* | Downstream (within SERPINB10) | 18 | 61,587,067 | rs9967382 | -0.107 | 0.222 |

^1^Amino acid changes indicated in parentheses for exonic missense and synonymous SNPs.

^2^Chromosomal positions refer to Human Genome Build 37.

^3^Beta coefficients with reference to minor alleles.

**Supplementary Table S5. Means and medians of gestational age for infants with each genotype for the *CPPED1* SNPs associated with gestational age.**

| **SNP** | **Genotype** | **Number of infants with the genotype** | **Mean / median ± standard deviation of gestational age (weeks)** |
| --- | --- | --- | --- |
| rs11643593 | AA | 68 (19.9%) | 40.23 / 40.29 ± 0.88 |
|  | CA | 180 (52.8%) | 40.19 / 40.29 ± 0.91 |
|  | CC | 93 (27.3%)) | 39.74 / 39.57 ± 0.83 |
| rs8048866 | GG | 23 (6.8%) | 39.81 / 39.71 ± 0.86 |
|  | GA | 130 (38.2%) | 39.89 / 40.00 ± 0.89 |
|  | AA | 187 (55.0%) | 40.24 / 40.43 ± 0.90 |

**Supplementary Table S6. Downregulated genes after CPPED1 silencing in HTR8/SVneo cells.** *CPPED1* was silenced using siRNAs and compared to the cells treated with sham siRNAs. Thresholds used in filtering the differentially expressed genes were: FC > 2.0 and FDR-adjusted *p*-value < 0.05. The differentially expressed genes have been ranked based both on *p*-value and fold change. For example, the first gene in the table (*CXCL8*) has the highest possible rank.

| **Gene name** | **FC^1^** | ***p*-value^2^** | **adj. *p*-value^3^** | **EntrezID** | **Description** |
| --- | --- | --- | --- | --- | --- |
| *CXCL8* | -6.133 | 0.000145 | 0.032258 | 3576 | C-X-C motif chemokine ligand 8 |
| *RCAN2* | -7.14 | 0.000352 | 0.012346 | 10231 | regulator of calcineurin 2 |
| *CEMIP* | -4.321 | 0.000137 | 0.033333 | 57214 | cell migration inducing hyaluronan binding protein |
| *CLCA2* | -5.452 | 0.000333 | 0.013158 | 9635 | chloride channel accessory 2 |
| *UBD* | -4.905 | 0.000341 | 0.012821 | 10537 | ubiquitin D |
| *CXCL1* | -3.68 | 0.000172 | 0.027778 | 2919 | C-X-C motif chemokine ligand 1 |
| *CHI3L2* | -8.236 | 0.000937 | 0.025773 | 1117 | chitinase 3 like 2 |
| *IL6* | -3.584 | 0.000113 | 0 | 3569 | interleukin 6 |
| *INPP5D* | -4.67 | 0.000655 | 0.013793 | 3635 | inositol polyphosphate-5-phosphatase D |
| *CPPED1* | -3.431 | 0.00000393 | 0 | 55313 | calcineurin like phosphoesterase domain containing 1 |
| *CCL2* | -3.639 | 0.000266 | 0.016949 | 6347 | C-C motif chemokine ligand 2 |
| *IL4I1* | -3.193 | 0.000383 | 0.011364 | 259307 | interleukin 4 induced 1 |
| *CCL7* | -4.133 | 0.001509 | 0.031847 | 6354 | C-C motif chemokine ligand 7 |
| *IL1B* | -3.15 | 0.000617 | 0.014493 | 3553 | interleukin 1 beta |
| *ROBO4* | -2.938 | 0.000511 | 0.008621 | 54538 | roundabout guidance receptor 4 |
| *ISLR* | -3.934 | 0.002011 | 0.029925 | 3671 | immunoglobulin superfamily containing leucine rich repeat |
| *TRIM22* | -3.039 | 0.001066 | 0.026906 | 10346 | tripartite motif containing 22 |
| *GBP2* | -3.453 | 0.001806 | 0.030641 | 2634 | guanylate binding protein 2 |
| *GDF15* | -3.186 | 0.001357 | 0.032143 | 9518 | growth differentiation factor 15 |
| *CEACAM1* | -3.089 | 0.001252 | 0.034483 | 634 | carcinoembryonic antigen related cell adhesion molecule 1 |
| *ELF3* | -3.004 | 0.001225 | 0.035433 | 1999 | E74 like ETS transcription factor 3 |
| *LINC01021* | -6.159 | 0.00352 | 0.04271 | 643401 | long intergenic non-protein coding RNA 1021 |
| *CXCL2* | -2.964 | 0.001934 | 0.03125 | 2920 | C-X-C motif chemokine ligand 2 |
| *AREG* | -3.49 | 0.002879 | 0.03777 | 374 | amphiregulin |
| *TNFRSF9* | -2.572 | 0.000777 | 0.024096 | 3604 | TNF receptor superfamily member 9 |
| *IL24* | -3.788 | 0.003705 | 0.043165 | 11009 | interleukin 24 |
| *IL1R1* | -2.288 | 0.000125 | 0.037037 | 3554 | interleukin 1 receptor type 1 |
| *ERC2* | -2.86 | 0.002423 | 0.033126 | 26059 | ELKS/RAB6-interacting/CAST family member 2 |
| *IL1A* | -3.091 | 0.003081 | 0.043993 | 3552 | interleukin 1 alpha |
| *PTGS2* | -3.263 | 0.00341 | 0.042748 | 5743 | prostaglandin-endoperoxide synthase 2 |
| *ABHD17C* | -2.207 | 0.0000544 | 0 | 58489 | abhydrolase domain containing 17C |
| *CLDN4* | -2.64 | 0.002186 | 0.032258 | 1364 | claudin 4 |
| *CALML6* | -3.359 | 0.004226 | 0.045337 | 163688 | calmodulin like 6 |
| *CXCL6* | -2.481 | 0.00186 | 0.032609 | 6372 | C-X-C motif chemokine ligand 6 |
| *CDH5* | -2.271 | 0.000789 | 0.023669 | 1003 | cadherin 5 |
| *PIK3CG* | -2.469 | 0.001942 | 0.031088 | 5294 | phosphatidylinositol-4.5-bisphosphate 3-kinase catalytic subunit gamma |
| *SAA2* | -3.126 | 0.004408 | 0.044944 | 6289 | serum amyloid A2 |
| *TNFAIP3* | -2.118 | 0.0000348 | 0 | 7128 | TNF alpha induced protein 3 |
| *PROM2* | -2.144 | 0.000246 | 0.018519 | 150696 | prominin 2 |
| *SAA1* | -2.598 | 0.00286 | 0.038043 | 6288 | serum amyloid A1 |
| *SERPINB4* | -5.743 | 0.006343 | 0.046737 | 6318 | serpin family B member 4 |
| *SNORD116-18* | -2.754 | 0.003897 | 0.048023 | 100033430 | small nucleolar RNA. C/D box 116-18 |
| *TIMP4* | -2.366 | 0.002229 | 0.031532 | 7079 | TIMP metallopeptidase inhibitor 4 |
| *NFAM1* | -2.993 | 0.004665 | 0.043581 | 150372 | NFAT activating protein with ITAM motif 1 |
| *MIR3613* | -2.754 | 0.003897 | 0.048091 | 100500908 | microRNA 3613 |
| *MIR6732* | -2.754 | 0.003897 | 0.048295 | 102465438 | microRNA 6732 |
| *NFKBIZ* | -2.166 | 0.000734 | 0.025316 | 64332 | NFKB inhibitor zeta |
| *CD68* | -2.15 | 0.000594 | 0.014925 | 968 | CD68 molecule |
| *LINCR-0001* | -3.325 | 0.005324 | 0.045643 | 101929191 | uncharacterized LINCR-0001 |
| *KCNN4* | -2.125 | 0.000586 | 0.015038 | 3783 | potassium calcium-activated channel subfamily N member 4 |
| *PLEKHG4* | -2.14 | 0.00103 | 0.027907 | 25894 | pleckstrin homology and RhoGEF domain containing G4 |
| *FCRLA* | -3.31 | 0.005819 | 0.047893 | 84824 | Fc receptor like A |
| *SERPINA3* | -4.724 | 0.007023 | 0.04956 | 12 | serpin family A member 3 |
| *RASGRF2* | -2.319 | 0.002725 | 0.037736 | 5924 | Ras protein specific guanine nucleotide releasing factor 2 |
| *TIE1* | -2.143 | 0.001341 | 0.032491 | 7075 | tyrosine kinase with immunoglobulin like and EGF like domains 1 |
| *VCAM1* | -3.355 | 0.006381 | 0.046491 | 7412 | vascular cell adhesion molecule 1 |
| *KRT15* | -3.824 | 0.006894 | 0.049755 | 3866 | keratin 15 |
| *SNORA77* | -3.351 | 0.00646 | 0.046794 | 677843 | small nucleolar RNA. H/ACA box 77 |
| *CDKN1A* | -2.045 | 0.000438 | 0.01 | 1026 | cyclin dependent kinase inhibitor 1A |
| *PTGES* | -2.073 | 0.001213 | 0.035857 | 9536 | prostaglandin E synthase |
| *LOC100128361* | -2.072 | 0.00149 | 0.032362 | 100128361 | uncharacterized LOC100128361 |
| *IP6K3* | -2.252 | 0.003422 | 0.042553 | 117283 | inositol hexakisphosphate kinase 3 |
| *ITGA10* | -2.095 | 0.002354 | 0.033898 | 8515 | integrin subunit alpha 10 |
| *SLC37A2* | -2.18 | 0.00345 | 0.042105 | 219855 | solute carrier family 37 member 2 |
| *TSHZ2* | -2.29 | 0.004828 | 0.044318 | 128553 | teashirt zinc finger homeobox 2 |
| *S100A3* | -2.229 | 0.005212 | 0.04661 | 6274 | S100 calcium binding protein A3 |
| *NLRP3* | -2.171 | 0.005281 | 0.046122 | 114548 | NLR family pyrin domain containing 3 |
| *PTPN22* | -2.104 | 0.005002 | 0.043908 | 26191 | protein tyrosine phosphatase. non-receptor type 22 |
| *NCKAP1L* | -2.235 | 0.006629 | 0.048346 | 3071 | NCK associated protein 1 like |
| *TRAF1* | -2.146 | 0.006254 | 0.047406 | 7185 | TNF receptor associated factor 1 |

^1^Expression ratio (fold-change) between the compared sample groups. The comparison was done between CPPED1 silenced cells and negative control cells.

^2^*T*-test *p*-value for the comparison between the sample groups (CPPED1 silenced and negative control cells)

^3^FDR-adjusted p-value

**Supplementary Table S7. Upregulated genes after *CPPED1* silencing in HTR8/SVneo cells.** *CPPED1* was silenced in HTR8/SVneo cells using siRNAs and compared to the cells treated with sham siRNAs. Thresholds used in filtering the differentially expressed genes were: FC > 2.0 and FDR-adjusted *p*-value < 0.05. The differentially expressed genes have been ranked based both on *p*-value and fold change. For example, the first gene in the table (*TYRP1*) has the highest possible rank.

| **Gene name** | **FC^1^** | ***p*-value^2^** | **adj. *p*-value^3^** | **EntrezID** | **Description** |
| --- | --- | --- | --- | --- | --- |
| *TYRP1* | 6.918 | 0.0000387 | 0 | 7306 | tyrosinase related protein 1 |
| *OLFML1* | 6.521 | 0.0000937 | 0 | 283298 | olfactomedin like 1 |
| *MFAP5* | 4.728 | 0.000376 | 0.011494 | 8076 | microfibrillar associated protein 5 |
| *PLCE1-AS1* | 4.175 | 0.000274 | 0.016393 | 100128054 | PLCE1 antisense RNA 1 |
| *SESN3* | 3.355 | 0.000636 | 0.014085 | 143686 | sestrin 3 |
| *MIR3607* | 3.85 | 0.001264 | 0.034091 | 100500805 | microRNA 3607 |
| *KIAA0226L* | 4.352 | 0.001588 | 0.033333 | NA | NA |
| *SEMA3E* | 5.278 | 0.001899 | 0.031915 | 9723 | semaphorin 3E |
| *HECTD2-AS1* | 7.773 | 0.00207 | 0.029056 | 100188947 | HECTD2 antisense RNA 1 |
| *KCND3* | 3.138 | 0.000559 | 0.015748 | 3752 | potassium voltage-gated channel subfamily D member 3 |
| *ANKRD30A* | 7.777 | 0.00219 | 0.032184 | 91074 | ankyrin repeat domain 30A |
| *GFRA1* | 3.217 | 0.000862 | 0.027473 | 2674 | GDNF family receptor alpha 1 |
| *VLDLR-AS1* | 3.096 | 0.000754 | 0.024691 | 401491 | VLDLR antisense RNA 1 |
| *JPH1* | 2.746 | 0.0000196 | 0 | 56704 | junctophilin 1 |
| *B3GALT2* | 4.913 | 0.002434 | 0.03299 | 8707 | beta-1.3-galactosyltransferase 2 |
| *NPR3* | 2.699 | 0.000235 | 0.019608 | 4883 | natriuretic peptide receptor 3 |
| *ANKRD1* | 2.76 | 0.000449 | 0.009709 | 27063 | ankyrin repeat domain 1 |
| *KCNB1* | 3.271 | 0.001568 | 0.033846 | 3745 | potassium voltage-gated channel subfamily B member 1 |
| *VSTM1* | 3.744 | 0.002458 | 0.032587 | 284415 | V-set and transmembrane domain containing 1 |
| *FRAS1* | 3.666 | 0.002442 | 0.032854 | 80144 | Fraser extracellular matrix complex subunit 1 |
| *SNORD56* | 3.207 | 0.002284 | 0.032895 | 26793 | small nucleolar RNA. C/D box 56 |
| *GJA5* | 2.358 | 0.0000623 | 0 | 2702 | gap junction protein alpha 5 |
| *SVEP1* | 2.379 | 0.00016 | 0.029412 | 79987 | sushi. von Willebrand factor type A. EGF and pentraxin domain containing 1 |
| *CPB1* | 3.576 | 0.003033 | 0.044905 | 1360 | carboxypeptidase B1 |
| *HIST2H2AB* | 4.431 | 0.003693 | 0.043353 | 317772 | histone cluster 2 H2A family member b |
| *MIR6734* | 3.92 | 0.003434 | 0.042489 | 102466723 | microRNA 6734 |
| *TARID* | 3.756 | 0.003501 | 0.042963 | 100507308 | TCF21 antisense RNA inducing promoter demethylation |
| *NPPB* | 2.296 | 0.000242 | 0.018868 | 4879 | natriuretic peptide B |
| *PPME1* | 2.264 | 0.000372 | 0.011628 | 51400 | protein phosphatase methylesterase 1 |
| *OLR1* | 2.241 | 0.0000780 | 0 | 4973 | oxidized low density lipoprotein receptor 1 |
| *KCNH1* | 2.339 | 0.00096 | 0.030303 | 3756 | potassium voltage-gated channel subfamily H member 1 |
| *TNNT2* | 3.746 | 0.004443 | 0.044499 | 7139 | troponin T2. cardiac type |
| *GLP2R* | 2.511 | 0.001731 | 0.031429 | 9340 | glucagon like peptide 2 receptor |
| *PRICKLE1* | 2.334 | 0.001232 | 0.035156 | 144165 | prickle planar cell polarity protein 1 |
| *ARHGAP28* | 2.201 | 0.000305 | 0.014493 | 79822 | Rho GTPase activating protein 28 |
| *PLEKHG7* | 8.467 | 0.005815 | 0.047939 | 440107 | pleckstrin homology and RhoGEF domain containing G7 |
| *FOLR1* | 2.572 | 0.002383 | 0.033755 | 2348 | folate receptor 1 |
| *PKP2* | 2.132 | 0.0000270 | 0 | 5318 | plakophilin 2 |
| *ALPK2* | 2.327 | 0.001887 | 0.032172 | 115701 | alpha kinase 2 |
| *SFTA1P* | 2.887 | 0.00411 | 0.046791 | 207107 | surfactant associated 1. pseudogene |
| *TMEM178A* | 2.27 | 0.001957 | 0.030848 | 130733 | transmembrane protein 178A |
| *CXADR* | 2.11 | 0.0005 | 0.00885 | 1525 | coxsackie virus and adenovirus receptor |
| *LAMA4* | 2.118 | 0.000674 | 0.013333 | 3910 | laminin subunit alpha 4 |
| *CTSF* | 3.16 | 0.005339 | 0.045455 | 8722 | cathepsin F |
| *COL2A1* | 2.395 | 0.003061 | 0.044369 | 1280 | collagen type II alpha 1 chain |
| *UCA1* | 4.205 | 0.006726 | 0.048536 | 652995 | urothelial cancer associated 1 (non-protein coding) |
| *C10orf62* | 2.736 | 0.004463 | 0.044335 | 414157 | chromosome 10 open reading frame 62 |
| *PSG3* | 6.49 | 0.007463 | 0.049624 | 5671 | pregnancy specific beta-1-glycoprotein 3 |
| *ACKR3* | 4.866 | 0.007313 | 0.048967 | 57007 | atypical chemokine receptor 3 |
| *PSG4* | 2.036 | 0.000628 | 0.014286 | 5672 | pregnancy specific beta-1-glycoprotein 4 |
| *NRK* | 2.115 | 0.001678 | 0.031977 | 203447 | Nik related kinase |
| *IL7R* | 2.128 | 0.001883 | 0.032258 | 3575 | interleukin 7 receptor |
| *SEMA3D* | 2.256 | 0.003211 | 0.042414 | 223117 | semaphorin 3D |
| *LAD1* | 2.97 | 0.006281 | 0.047237 | 3898 | ladinin 1 |
| *COL8A1* | 2.139 | 0.002126 | 0.033254 | 1295 | collagen type VIII alpha 1 chain |
| *DTX4* | 2.034 | 0.000582 | 0.015152 | 23220 | deltex E3 ubiquitin ligase 4 |
| *NRXN3* | 2.092 | 0.001965 | 0.030691 | 9369 | neurexin 3 |
| *TERT* | 2.387 | 0.004762 | 0.045035 | 7015 | telomerase reverse transcriptase |
| *TMEM255A* | 2.13 | 0.002493 | 0.032064 | 55026 | transmembrane protein 255A |
| *EDNRA* | 2.059 | 0.001795 | 0.030812 | 1909 | endothelin receptor type A |
| *TMEM52B* | 2.508 | 0.00625 | 0.047449 | 120939 | transmembrane protein 52B |
| *EDN1* | 2.343 | 0.005689 | 0.049213 | 1906 | endothelin 1 |
| *RAD21-AS1* | 2.117 | 0.003481 | 0.041729 | 644660 | RAD21 antisense RNA 1 |
| *DIO2* | 2.105 | 0.003748 | 0.04578 | 1734 | deiodinase. iodothyronine type II |
| *CPA1* | 2.332 | 0.006215 | 0.047791 | 1357 | carboxypeptidase A1 |
| *SLIT3* | 2.045 | 0.003088 | 0.043845 | 6586 | slit guidance ligand 3 |
| *LOC102724596* | 2.088 | 0.004431 | 0.044665 | 102724596 | uncharacterized LOC102724596 |
| *IL18* | 2.144 | 0.004979 | 0.042904 | 3606 | interleukin 18 |
| *ANXA3* | 2.243 | 0.006168 | 0.048182 | 306 | annexin A3 |
| *PIK3IP1* | 2.279 | 0.00666 | 0.048142 | 113791 | phosphoinositide-3-kinase interacting protein 1 |
| *IGFBP5* | 2.026 | 0.003375 | 0.043344 | 3488 | insulin like growth factor binding protein 5 |
| *UCP3* | 2.318 | 0.00712 | 0.049606 | 7352 | uncoupling protein 3 |
| *NETO1* | 2.277 | 0.007097 | 0.049763 | 81832 | neuropilin and tolloid like 1 |
| *ADAMTS5* | 2.242 | 0.006664 | 0.048101 | 11096 | ADAM metallopeptidase with thrombospondin type 1 motif 5 |
| *ITGB8* | 2.012 | 0.004345 | 0.044248 | 3696 | integrin subunit beta 8 |
| *SYNPO2* | 2.018 | 0.005424 | 0.044715 | 171024 | synaptopodin 2 |
| *SNORD21* | 2.021 | 0.00689 | 0.049796 | 6083 | small nucleolar RNA. C/D box 21 |

^1^Expression ratio (fold-change) between the compared sample groups. The comparison was done between CPPED1 silenced cells and negative control cells.

^2^*T*-test *p*-value for the comparison between the sample groups (CPPED1 silenced and negative control cells)

^3^FDR-adjusted p-value

**Supplementary Table S8. Functional classification of differentially expressed genes after post-transcriptional silencing of CPPED1 in HTR8/SVneo cells.** Clustering is according to Gene Ontology (GO) biological processes and molecular functions. Thirty most significant GO terms in each category are shown

| **GO ID** | **GO Term** | **Total amount of annotated genes** | **The amount of significant genes** | ***p*-value** |
| --- | --- | --- | --- | --- |
| **Biological process** | | | | |
| GO:0001568 | blood vessel development | 570 | 25 | 4.2 × 10^-13^ |
| GO:0032496 | response to lipopolysaccharide | 295 | 19 | 4.8 × 10^-13^ |
| GO:0006954 | inflammatory response | 643 | 26 | 8.5 × 10^-13^ |
| GO:0001944 | vasculature development | 591 | 25 | 9.3 × 10^-13^ |
| GO:0002237 | response to molecule of bacterial origin | 308 | 19 | 1.0 × 10^-12^ |
| GO:0072358 | cardiovascular system development | 598 | 25 | 1.2 × 10^-12^ |
| GO:0034097 | response to cytokine | 777 | 28 | 1.6 × 10^-12^ |
| GO:1990266 | neutrophil migration | 87 | 12 | 1.6 × 10^-12^ |
| GO:0072359 | circulatory system development | 909 | 30 | 2.0 × 10^-12^ |
| GO:0001525 | angiogenesis | 410 | 21 | 2.1 × 10^-12^ |
| GO:0006952 | defense response | 1433 | 37 | 4.6 × 10^-12^ |
| GO:0048514 | blood vessel morphogenesis | 486 | 22 | 7.0 × 10^-12^ |
| GO:0016477 | cell migration | 1180 | 33 | 1.1 × 10^-11^ |
| GO:0097530 | granulocyte migration | 105 | 12 | 1.6 × 10^-11^ |
| GO:0051270 | regulation of cellular component movement | 764 | 26 | 4.1 × 10^-11^ |
| GO:0019221 | cytokine-mediated signaling pathway | 540 | 22 | 5.4 × 10^-11^ |
| GO:0040011 | locomotion | 1482 | 36 | 5.7 × 10^-11^ |
| GO:0030334 | regulation of cell migration | 659 | 24 | 6.5 × 10^-11^ |
| GO:0097529 | myeloid leukocyte migration | 149 | 13 | 6.8 × 10^-11^ |
| GO:0009617 | response to bacterium | 497 | 21 | 8.0 × 10^-11^ |
| GO:0071345 | cellular response to cytokine stimulus | 683 | 24 | 1.4 × 10^-10^ |
| GO:0050900 | leukocyte migration | 360 | 18 | 1.4 × 10^-10^ |
| GO:0048870 | cell motility | 1298 | 33 | 1.4 × 10^-10^ |
| GO:0051674 | localization of cell | 1298 | 33 | 1.4 × 10^-10^ |
| GO:0033993 | response to lipid | 824 | 26 | 2.1 × 10^-10^ |
| GO:2000145 | regulation of cell motility | 707 | 24 | 2.7 × 10^-10^ |
| GO:0030593 | neutrophil chemotaxis | 81 | 10 | 3.8 × 10^-10^ |
| GO:0040012 | regulation of locomotion | 736 | 24 | 6.1 × 10^-10^ |
| GO:0009605 | response to external stimulus | 1964 | 40 | 7.3 × 10^-10^ |
| GO:0006928 | movement of cell or subcellular component | 1725 | 37 | 9.8 × 10^-10^ |
| **Molecular function** | | | | |
| GO:0005125 | cytokine activity | 220 | 15 | 8.9 × 10^-11^ |
| GO:0005102 | receptor binding | 1400 | 32 | 5.7 × 10^-9^ |
| GO:0008009 | chemokine activity | 48 | 6 | 1.4 × 10^-6^ |
| GO:0005126 | cytokine receptor binding | 266 | 11 | 4.8 × 10^-6^ |
| GO:0042379 | chemokine receptor binding | 60 | 6 | 5.4 × 10^-6^ |
| GO:0045236 | CXCR chemokine receptor binding | 17 | 4 | 6.5 × 10^-6^ |
| GO:0005539 | glycosaminoglycan binding | 204 | 8 | 1.4 × 10^-4^ |
| GO:0001664 | G-protein coupled receptor binding | 254 | 8 | 6.3 × 10^-4^ |
| GO:0008201 | heparin binding | 158 | 6 | 1.2 × 10^-3^ |
| GO:0044325 | ion channel binding | 111 | 5 | 1.4 × 10^-3^ |
| GO:1901681 | sulfur compound binding | 228 | 7 | 1.6 × 10^-3^ |
| GO:0070851 | growth factor receptor binding | 125 | 5 | 2.4 × 10^-3^ |
| GO:0004896 | cytokine receptor activity | 89 | 4 | 4.4 × 10^-3^ |
| GO:0019903 | protein phosphatase binding | 108 | 4 | 8.7 × 10^-3^ |
| GO:0002020 | protease binding | 112 | 4 | 9.8 × 10^-3^ |
| GO:0005515 | protein binding | 8753 | 78 | 1.1 × 10^-2^ |
| GO:0005267 | potassium channel activity | 119 | 4 | 1.2 × 10^-2^ |
| GO:0038024 | cargo receptor activity | 66 | 3 | 1.3 × 10^-2^ |
| GO:0008528 | G-protein coupled peptide receptor activity | 128 | 4 | 1.5 × 10^-2^ |
| GO:0001653 | peptide receptor activity | 129 | 4 | 1.6 × 10^-2^ |
| GO:0005201 | extracellular matrix structural constituent | 77 | 3 | 2.0 × 10^-2^ |
| GO:0005089 | Rho guanyl-nucleotide exchange factor activity | 78 | 3 | 2.1 × 10^-2^ |
| GO:0004872 | receptor activity | 1585 | 19 | 2.5 × 10^-2^ |
| GO:0060089 | molecular transducer activity | 1585 | 19 | 2.5 × 10^-2^ |
| GO:0019902 | phosphatase binding | 150 | 4 | 2.6 × 10^-2^ |
| GO:0015079 | potassium ion transmembrane transporter activity | 151 | 4 | 2.6 × 10^-2^ |
| GO:0005249 | voltage-gated potassium channel activity | 88 | 3 | 2.8 × 10^-2^ |
| GO:0019955 | cytokine binding | 91 | 3 | 3.0 × 10^-2^ |
| GO:0008083 | growth factor activity | 161 | 4 | 3.2 × 10^-2^ |
| GO:0005178 | integrin binding | 102 | 3 | 4.1 × 10^-2^ |

**FIGURE LEGENDS**

**Figure S1. Representative 2D gel of human placenta tissue after spontaneous term birth.** Placenta proteins (50 μg) collected from the basal plate of the placenta were labeled with Cy5 (minimal difference gel electrophoresis) and separated by isoelectic focusing (pH4–7, 24 cm) and SDS-PAGE. Positions of spots that were significantly changed are indicated.

**Figure S2. The effect of siRNA on *CPPED1* mRNA levels.** CPPED1 was post-transcriptionally silenced in HTR8/SVneo cells that is a human placental trophoblast continuous cell line. RNA was isolated from *CPPED1* silenced cells and compared to RNA from control cells. In the figure, *CPPED1* expression levels are shown as determined by qRT-PCR (A) and high throughput RNA sequencing (B). Relative mRNA levels were normalized to mRNA levels of the housekeeping gene *CYC1* (A). Reads per kilobase of exon per million reads mapped (RKPM) is a normalized gene counts value determined in the transcriptomic analysis (B). The columns show the mean value of triplicate samples; the maximum and minimum values are also indicated.

**FIGURES**

**Figure S1**

**
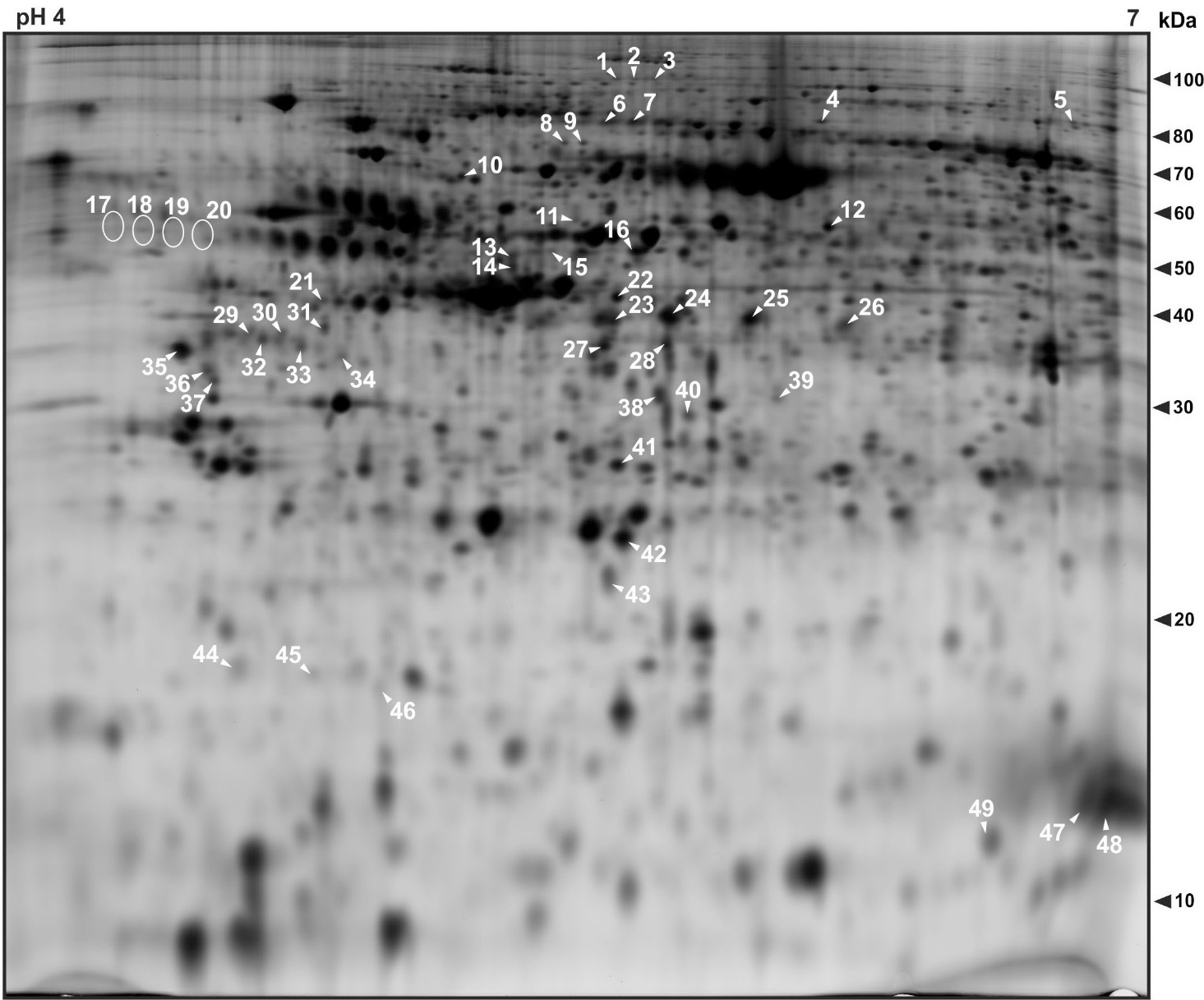
**

**Figure S2**

**
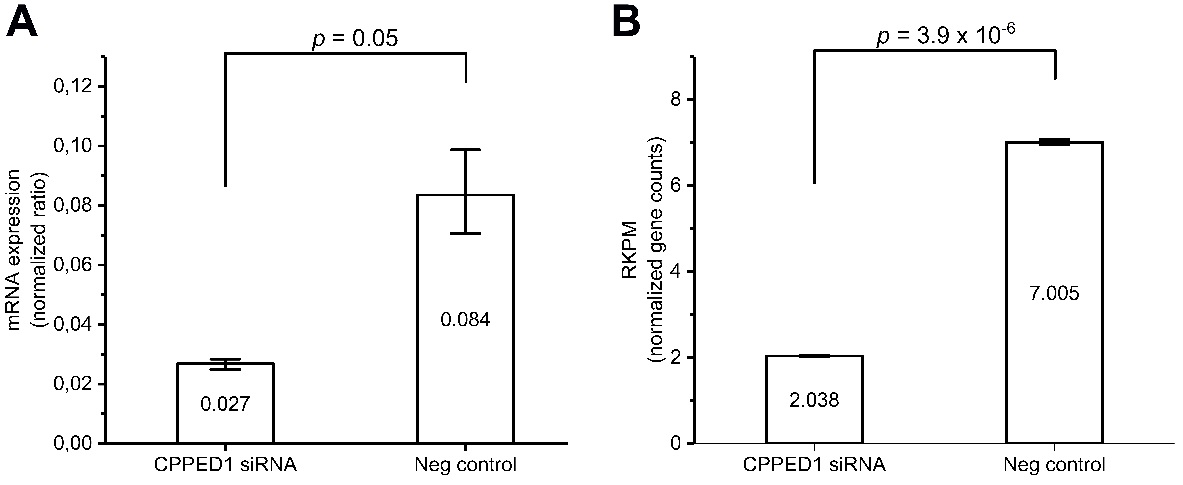
**
