# Supplementary figures and images for: Expression of CPPED1 in human trophoblasts is associated with timing of term birth
Source: J Cell Mol Med. 2017 Nov 29;22(2):968–81. doi: 10.1111/jcmm.13402 (PMC5783879; doi:10.1111/jcmm.13402)

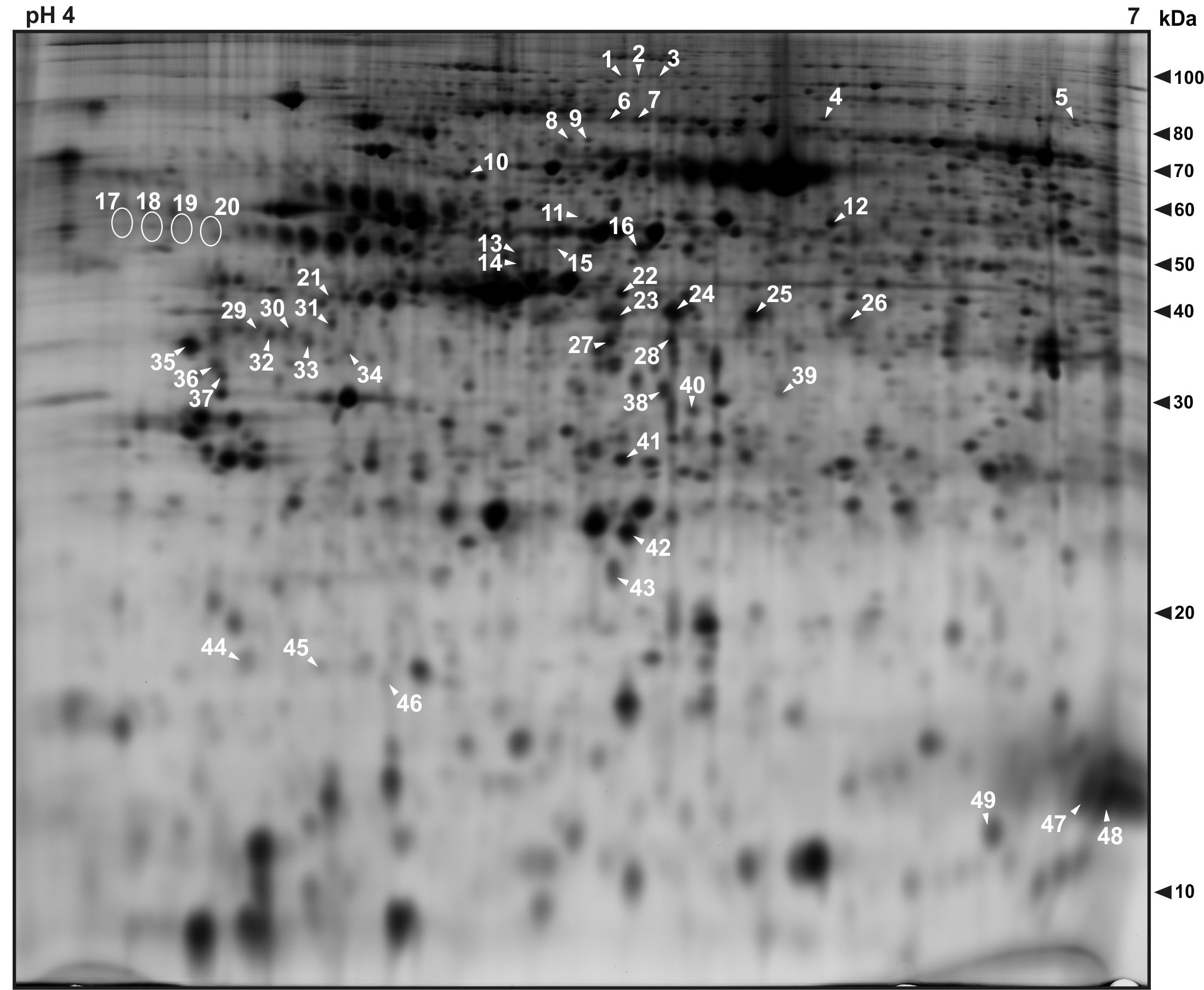

Supplement: Supplementary file 2 — Figure S1 Representative 2D gel of human placenta tissue after spontaneous term birth. Placenta proteins (50 μg) collected from the basal plate of the placenta were labeled with Cy5 (minimal difference gel electrophoresis) and separated by isoelectic focusing (pH4–7, 24 cm) and SDS‐PAGE. Positions of spots that were significantly changed are indicated. [file JCMM-22-968-s002.tif]

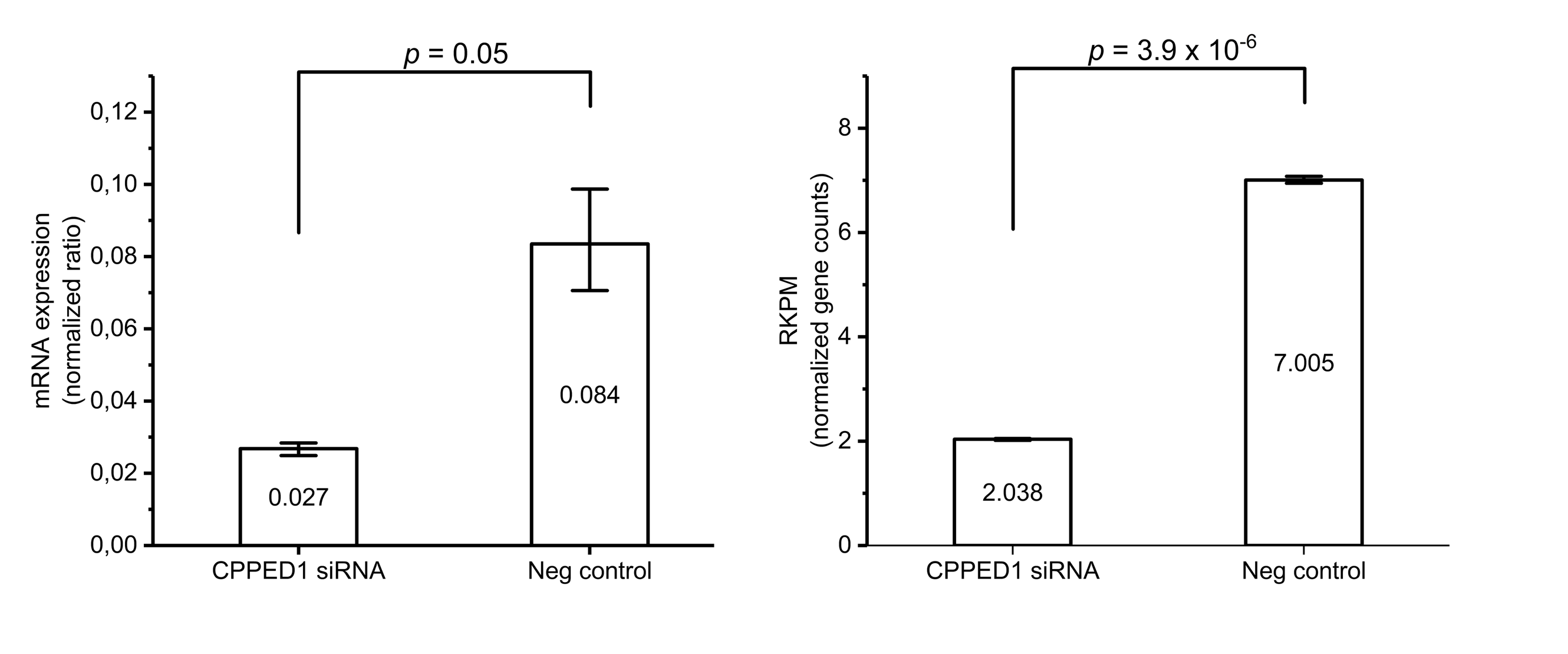

Supplement: Supplementary file 3 — Figure S2 The effect of siRNA on CPPED1 mRNA levels. CPPED1 was post‐transcriptionally silenced in HTR8/SVneo cells that is a human placental trophoblast continuous cell line. RNA was isolated from CPPED1 silenced cells and compared to RNA from control cells. In the figure, CPPED1 expression levels are shown as determined by qRT‐PCR (A) and high throughput RNA sequencing (B). Relative mRNA levels were normalized to mRNA levels of the housekeeping gene CYC1 (A). Reads per kilobase of exon per million reads mapped (RKPM) is a normalized gene counts value determined in the transcriptomic analysis (B). The columns show the mean value of triplicate samples; the maximum and minimum values are also indicated. [file JCMM-22-968-s003.tif]
